# Understanding the role of the NMDA receptor subunit, GluN2D, in mediating NMDA receptor antagonist‐induced behavioral disruptions in male and female mice

**DOI:** 10.1002/jnr.25257

**Published:** 2023-10-10

**Authors:** Chitra Vinnakota, Anna Schroeder, Xin Du, Kazutaka Ikeda, Soichiro Ide, Masayoshi Mishina, Matthew Hudson, Nigel Charles Jones, Suresh Sundram, Rachel Anne Hill

**Affiliations:** ^1^ Department of Psychiatry Monash University Clayton Victoria Australia; ^2^ Addictive Substance Project Tokyo Metropolitan Institute of Medical Science Tokyo Japan; ^3^ Brain Science Laboratory, The Research Organization of Science and Technology Ritsumeikan University Kusatsu Japan; ^4^ Department of Neuroscience Monash University Clayton Victoria Australia; ^5^ Mental Health Program Monash Health Clayton Victoria Australia

**Keywords:** GluN2D, ketamine, mouse behaviour, NMDA, PCP, schizophrenia

## Abstract

Noncompetitive NMDA receptor (NMDAR) antagonists like phencyclidine (PCP) and ketamine cause psychosis‐like symptoms in healthy humans, exacerbate schizophrenia symptoms in people with the disorder, and disrupt a range of schizophrenia‐relevant behaviors in rodents, including hyperlocomotion. This is negated in mice lacking the GluN2D subunit of the NMDAR, suggesting the GluN2D subunit mediates the hyperlocomotor effects of these drugs. However, the role of GluN2D in mediating other schizophrenia‐relevant NMDAR antagonist‐induced behavioral disturbances, and in both sexes, is unclear. This study aimed to investigate the role of the GluN2D subunit in mediating schizophrenia‐relevant behaviors induced by a range of NMDA receptor antagonists. Using both male and female GluN2D knockout (KO) mice, we examined the effects of the NMDAR antagonist's PCP, the S‐ketamine enantiomer (S‐ket), and the ketamine metabolite R‐norketamine (R‐norket) on locomotor activity, anxiety‐related behavior, and recognition and short‐term spatial memory. GluN2D‐KO mice showed a blunted locomotor response to R‐norket, S‐ket, and PCP, a phenotype present in both sexes. GluN2D‐KO mice of both sexes showed an anxious phenotype and S‐ket, R‐norket, and PCP showed anxiolytic effects that were dependent on sex and genotype. S‐ket disrupted spatial recognition memory in females and novel object recognition memory in both sexes, independent of genotype. This datum identifies a role for the GluN2D subunit in sex‐specific effects of NMDAR antagonists and on the differential effects of the R‐ and S‐ket enantiomers.

## Graphical abstract

NMDA receptor antagonist R‐norket, S‐ket and PCP increase locomotor activity in WT but not GluNR2D KO mice. R‐norket causes anxiety like behaviours only in GluN2D KO mice. S‐Ket and PCPC increase anxiety in WT mice but have different effects in GluN2D KO mice depending on sex. S‐ket but not R‐norket or PCP disrupts spatial and episodic memory irrespective of genotype.

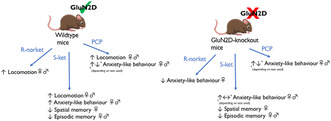

**Abbreviations**
EPM: elevated plus maze
i.p.: intraperitoneal
KO: knockout
NMDAR: N‐methyl‐d‐aspartate receptors
NOR: novel object recognition
OFT: open field test
PV: parvalbumin
PCP: phencyclidine
R‐ket: R‐ketamine
R‐norket: R‐norketamine
S‐ket: S‐ketamine
WT: wild type

**Significance**

These data provide significant new information that the sex of a mouse can influence how psychosis‐inducing drugs impact behaviors, including anxiety, locomotion, learning, and memory. This study finds that GluN2D, a specific subunit of a receptor complex, is required to mediate certain effects of psychosis‐inducing drugs and should be further explored as a novel therapeutic target. In particular, GluN2D mediates the effects of psychosis‐inducing drugs on locomotor‐ and anxiety‐related behaviors but not learning and memory. This has significant implications when considering how males and females manifest psychotic behaviors and whether treatments for psychotic disorders should be sex specific.

## 1 INTRODUCTION

N‐methyl‐d‐aspartate receptors (NMDARs) are a glutamate receptor subtype that are widely distributed in the central nervous system and play a key role in many physiological processes including neurodevelopment, synaptogenesis, synaptic plasticity, and several forms of cognition (Driesen et al., 2013; Hansen et al., 2017; Paoletti et al., 2013; Traynelis et al., 2010). NMDARs are heterotetramers composed of two obligatory GluN1 subunits and two subunits from among the GluN2A‐D or GluN3A‐B subunits (Karakas & Furukawa, 2014; Traynelis et al., 2010). The different subunits that come together to form a functional receptor generate different functional and pharmacological properties (Wyllie et al., 2013). Each various subunit also has unique temporal, regional, and cell‐specific expression patterns. The obligatory GluN1 subunit is ubiquitously expressed in the brain over the lifespan whereas the GluN2A and GluN2B are the predominant subtypes in the adult brain and the GluN2C and GluN2D subunits are more highly expressed in the early stages of life (Akazawa et al., 1994; Henson et al., 2008; Monyer et al., 1994; Tolle et al., 1993).

With maturation, GluN2D subunit expression markedly decreases and becomes confined to specific brain cell types, namely interneurons, Golgi and stellate cells, within the cortex, diencephalon, mesencephalon, and brain stem (Akazawa et al., 1994; Alsaad et al., 2019; Engelhardt et al., 2015; Garst‐Orozco et al., 2020). The GluN2D subunit in the adult brain is especially enriched in parvalbumin (PV)‐containing interneurons, a cell population implicated in the generation of high‐frequency gamma neural oscillations which are thought to underlie higher order cognitive processes (Antonoudiou et al., 2020; Engelhardt et al., 2015; Garst‐Orozco et al., 2020; Hanson et al., 2019; Hudson et al., 2020; Perszyk et al., 2016). Both parvalbumin interneurons and gamma oscillations are consistently reported to be disrupted in schizophrenia (Chen et al., 2014; Chung et al., 2022; Dienel & Lewis, 2019; Enwright et al., 2016; Enwright III et al., 2018; Gonzalez‐Burgos et al., 2015; Kaar et al., 2019), and in addition, post‐mortem studies have reported alterations in the expression of the GluN2D subunit in the prefrontal cortex of people with schizophrenia (Akbarian et al., 1996).

NMDAR antagonists like phencyclidine (PCP) and ketamine have long been reported to have psychotomimetic effects leading to the hypothesis that NMDAR hypofunction is central to the etiology and pathophysiology of schizophrenia (Balu, 2016; Cohen et al., 2015; Nakazawa & Sapkota, 2020). Multiple studies have shown that administration of sub‐anesthetic doses of ketamine and PCP can induce a spectrum of schizophrenia‐like symptoms including psychosis and neurocognitive disturbances in healthy individuals, and exacerbate symptoms in people with schizophrenia (Allen & Young, 1978; Cheng et al., 2018; Krystal et al., 1994; Lahti et al., 1995; Luby et al., 1959; Xu et al., 2015). In rodents, administration of these NMDAR channel blockers results in hyperlocomotion (proposed to represent a striatal hyperdopaminergic state which is thought to underlie the positive symptoms of schizophrenia like psychosis), disruption of the prepulse inhibition of the startle response, and cognitive deficits as assessed by various behavioral tests including the T‐maze and object recognition tasks (Cadinu et al., 2018; Gigg et al., 2020; Neill et al., 2014; Plataki et al., 2021; Sahin et al., 2016; Suryavanshi et al., 2014; Usun et al., 2013). Multiple studies have reported that GluN2D knockout (KO) mice are resistant to NMDAR antagonist‐induced hyperlocomotion, thus suggesting a role for the GluN2D subunit in mediating psychotomimetic drug‐induced hyperlocomotor behavior (Hagino et al., 2010; Ikeda et al., 1995; Sapkota et al., 2016; Yamamoto et al., 2016). The role of the GluN2D subunit in mediating NMDAR antagonist‐induced alterations to cognitive and negative symptom domains is less clear.

Ketamine is a racemic mixture of equal amounts of the enantiomers R‐ and S‐ketamine (R‐Ket; S‐ket). R‐Ket and S‐ket are thought to have subtly different pharmacological properties and behavioral effects (Chang et al., 2019; Domino & Warner, 2010; Fukumoto et al., 2017; Rafało‐Ulińska & Pałucha‐Poniewiera, 2022; Zhang, Ye, et al., 2021). S‐ket is reported to have higher affinity for the NMDAR and greater analgesic and anesthetic properties and is thus often thought of as the more potent isomer (Ebert et al., 1997; Jelen et al., 2021; White et al., 1985). The Food and Drug Administration (FDA) and European Medicines Agency (EMA) recently approved low‐dose intranasal S‐ket for use in people with treatment‐resistant depression following findings from multiple clinical trials of rapid‐onset, robust anti‐depressant effects (Canuso et al., 2018; Daly et al., 2021; Singh et al., 2016; Turner, 2019). Other studies, however, suggest that R‐ket may have more clinical relevance as it is associated with fewer unwanted psychotomimetic or dissociative symptoms and thus might be better tolerated by patients (Fukumoto et al., 2017; Leal et al., 2021) and several preclinical studies report that R‐ket has longer lasting anti‐depressant effects (Scotton et al., 2022; Yang et al., 2015; Zhang et al., 2014). While multiple rodent studies have shown anxiolytic effects of acute RS‐ket at sub‐anesthetic doses (.1–15 mg/kg) (Camargo et al., 2021; Fraga et al., 2018; Hou et al., 2022), limited studies have investigated the differential effects and behavioral responses induced by the two enantiomers in mice. A better understanding of the role of specific NMDAR subunits, such as the GluN2D subunit, in mediating enantiomer‐specific effects is needed.

In regard to cognitive domains, a study by Ide et al. (2019) reported that while RS‐ket, R‐ket, and S‐ket all produced impairments to object recognition in wild‐type (WT) mice, GluN2D‐KO mice were resistant to the effects of R‐ket on object recognition performance (Ide et al., 2019). This suggests that the GluN2D subunit may play an important role in mediating the effects of R‐ket on object recognition but not necessarily S‐ or RS‐ket. Whether this extends to other cognitive tasks is unknown.

Another lesser examined factor is the potential sex differences in response to NMDAR antagonists. This is despite well‐known sex differences in schizophrenia incidence, and within the cognitive and negative symptom domains (Mendrek & Mancini‐Marie, 2016). While PCP and RS‐ket have been reported to have sexually dimorphic effects on anxiety and cognitive behaviors (Fitzgerald et al., 2021; Liang et al., 2014; Turgeon et al., 2011), the role of the GluN2D subunit in mediating the effects of PCP or the ketamine enantiomers (R‐ket or S‐ket) has not been thoroughly investigated in both sexes to date.

The aims of this study were, using WT and GluN2D‐KO mice, to determine the role of the GluN2D subunit in a range of schizophrenia‐relevant behaviors; examine if the psychotomimetic NMDAR antagonists PCP, R‐norket, and S‐ket elicited distinct responses; and identify if these showed sexual dimorphism.

## 2 METHODS

### 2.1 Animals and groups

Male and female GluN2D‐KO mice were transported from Tokyo Metropolitan Institute of Medical Science and a breeding colony was established and maintained at the Monash Animal Research Platform, Monash University (Clayton, Victoria Australia), whereby GluN2D heterozygous male and female mice were bred to obtain WT, heterozygous and homozygous GluN2D‐KO littermates. Two cohorts of mice were used for this study; Cohort 1 was used for all behavioral tests except for the locomotor scoring assay for which Cohort 2 was used. All husbandry, housing, and behavioral testing was undertaken at the Department of Psychiatry, School of Clinical Sciences, Monash University for Cohort 1 and at the Department of Neuroscience, Central Clinical School, Monash University for Cohort 2. All mice were housed in groups of two to five in individually ventilated cages (Tecniplast, NSW, Australia) with ad libitum access to food and water. At 6–7 weeks of age, mice were transferred from the breeding facility to the behavioral holding room with a reversed 12‐h dark–light cycle (lights off at 8:30 am) and kept there until the end of the experiment. Cages were monitored daily and changed fortnightly.

Male and female WT and GluN2D‐KO mice were used for the experiments. With Cohort 1, mice were randomly assigned to four treatment groups (8–12 mice per group): mice received either saline (control; group 1), or the NMDAR antagonists PCP (group 2), R‐norket (group 3), or S‐ket (group 4). Hence, 16 groups were used in total (2*sex × 2*genotype × 4*treatment). With Cohort 2, all mice (*n* = 42) randomly received all four treatments with at least 48 h between treatments. All procedures were approved by the Monash University Animal Ethics Committee and comply with the ARRIVE guidelines. For behavioral testing and drug challenge, researchers were blinded to the genotype of the mice but not the drug type.

### 2.2 NMDAR antagonists

S‐ket (S‐(+)‐ketamine hydrochloride, 4379/50, TOCRIS, Bristol, UK), the ketamine metabolite R‐norket (R‐norketamine hydrochloride, 5996/10, TOCRIS), and PCP (Phencyclidine hydrochloride, 2557/10, TOCRIS, UK) were dissolved in saline to the final concentration required for the doses 25, 25, and 3 mg/kg, respectively. Doses were chosen based on previous studies (Fukumoto et al., 2017; Hagino et al., 2010). Each compound was delivered via a single intraperitoneal (i.p.) injection at 5 μL/mg as described for each test.

### 2.3 Behavioral testing

**FIGURE 1:**
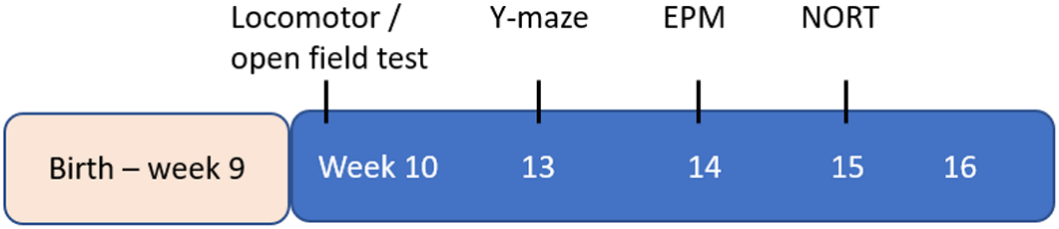
Timeline of behavioral testing. EPM, elevated plus maze; NORT, novel object recognition test.

Behavioral testing for Cohort 1 began at 10 weeks of age until ~16 weeks in the following order: locomotor test/open field test (OFT), spatial recognition memory test (Y‐maze), fear/anxiety‐related task, the elevated plus maze (EPM), and novel object recognition (NOR) task (see Figure 1 for timeline below). Behavioral testing for Cohort 2 was conducted at ~8–11 months. All behavioral tests were conducted during 9 am to 5 pm. At the end of experimentation, animals were humanely euthanized by cervical dislocation.

For data presentation, we categorized all behavioral tasks into three major groups according to modalities assessed: (1) Tasks related to locomotion, (2) fear/anxiety (OFT, EPM), and (3) novelty recognition memory (Y‐maze, NOR task). Body weight was analyzed at two time points: week 10, just before the experiments and week ~ 16 after completion of all behavioral tasks in Cohort 1 (Figure S1).

### 2.4 Locomotor test

Each mouse was placed into a 600 × 600 × 500 mm open field arena (SD Instruments, San Diego, USA) and the automated system recorded animal movement via infrared sensors. Baseline locomotor activity was measured for 1 h, after which the mice were injected with either saline, R‐norket (25 mg/kg), S‐ket (25 mg/kg), or PCP (3 mg/kg) according to their group allocation and locomotor activity was measured again for 2 h (Figure 1a) (Chavez et al., 2009; Klug et al., 2012; van den Buuse et al., 2012). The distance traveled was recorded during the entire 3‐h session and presented as total distance traveled during the first hour, average distance traveled during the last 2 h and additionally presented as 5‐min bins during the first hour and 20‐min bins during the last 2 h.

### 2.5 Locomotor scoring assay

While assessing behavior in the locomotor arenas following drug administration, we noted that altered locomotion may be due to a range of behaviors other than locomotion per se, including stereotypies, ataxia, and catalepsy. Thus, Cohort 2 was used to assess these behaviors. Mice were placed in a 370 × 190 × 130 mm open field arena and monitored visually for the presence of stereotypies, ataxia, and catalepsy for 30 min post‐injection. A score was given for each of the three measures every 5 min according to a 3‐point scale previously developed by our lab, and then, the score averaged over the 30‐min session (Hudson et al., 2020).

### 2.6 Tests to assess anxiety/fear‐related behavior

#### 2.6.1 Open field test

**FIGURE 2:**
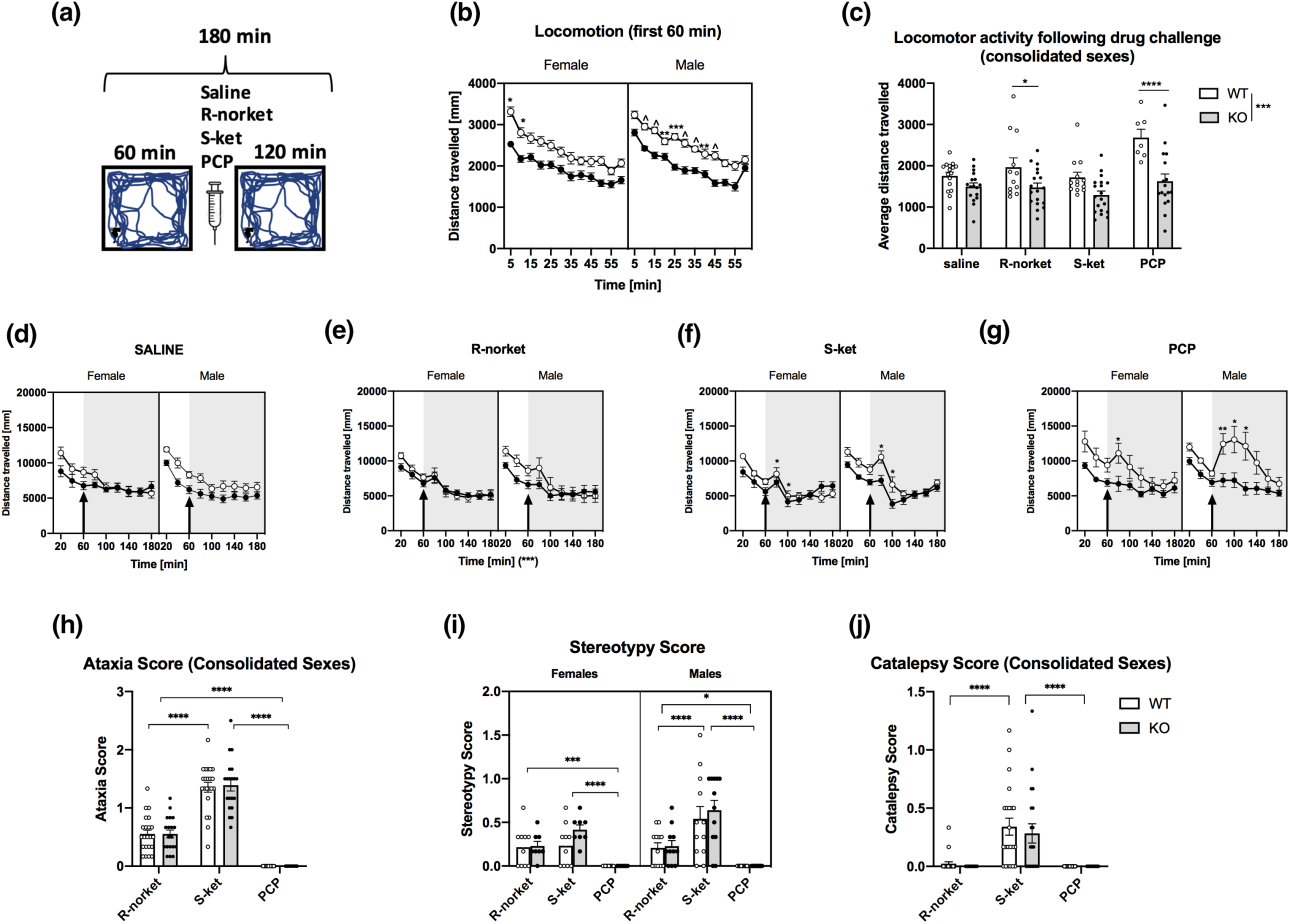
The effect of NMDAR antagonists on locomotion in WT and GluN2D‐KO mice. (a) Schematic overview of the paradigm. Locomotor activity without drug during the first 1 h (b), and locomotor activity over 2 h following drug challenge (c–g). Average distance traveled (c) and distance traveled over time with either saline (d; *n* = 9 F WT, 9 F KO, 9 M WT, 10 M KO), R‐norket (e; *n* = 10 F WT, 10 F KO, 10 M WT, 9 M KO), S‐ket (f; 9 F WT, 9 F KO, 10 M WT, 10 M KO), or PCP (g; 9 F WT, 10 F KO, 10 M WT, 11 M KO). Further measures of ataxia (h), stereotypy (i), and catalepsy (j) were assessed in a separate cohort (*n* = 10 F WT, 8 F KO, 12 M WT, 12 M KO). All data presented as mean ± SEM. When a two‐way ANOVA showed a significant interaction, *p*‐values were calculated with the Sidak's multiple comparison test (b, c), Fisher's LSD test (d–f, g: females), Tukey's multiple comparison test (g: males, h–j); *p < .05, **p < .01, ***p < .001, ****p < .0001.

Data from the above locomotor test were used to analyze anxiety‐like behavior as part of the OFT. The arena was divided into a center area (25% of arena) and four equal squared corners (combined, 25% of arena) (Figure 2a). The time spent in the center was calculated using the software PAS764 provided by SD Instruments. Less time spent in the center was taken to represent anxiety/fear‐related behavior.

#### 2.6.2 Elevated plus maze

EPM was performed as previously described (Grech et al., 2019). Saline, R‐norket, S‐ket, or PCP was administered 30 min before the test via a single i.p. injection. The level of anxiety‐like behavior was presented as time spent in the open arms over the first 5 min. A lower number (lower preference for the open arms) indicates a more anxious phenotype.

### 2.7 Tests to assess novel recognition memory

#### 2.7.1 Y‐maze—Novel‐arm recognition memory

Y‐maze was performed as previously described (Grech et al., 2019). Briefly, mice were placed in a Y‐shaped maze and allowed to explore two arms (1 arm closed) for 5 min. Mice were then removed from the maze and injected with saline, R‐norket, S‐ket, or PCP and returned to their home cage for 1 h. After the 1‐h retention period, mice were once again placed in the maze but this time with all three arms open and their activity was recorded for 5 min. The natural behavior of mice is to explore novel environments; therefore, intact recognition memory of the previously closed arm is reflected by an increased time spent in the novel arm upon reintroduction into the maze. The discrimination ratio was calculated as time spent in the novel arm/[(time spent on average in familiar arms) + (time spent in the novel arm)] (Ribeiro et al., 2019). A value above .5 indicates preference for the novel arm. Time spent in the center area (decision‐making phase) was analyzed separately. In this task, mice were exposed to NMDAR antagonists during the retention phase, therefore assessing the impact of the drugs on memory consolidation.

#### 2.7.2 Novel‐object recognition (NOR) task

NOR task was performed as previously described (Grech et al., 2019). After 2 days of habituation to the test apparatus, mice were injected with saline, R‐norket, S‐ket, or PCP 30 min before the test. The test began by exposing mice to two novel objects for 10 min. After 1‐h retention time, mice were exposed to one familiar object from the first phase and one novel object for 5 min. Objects were pseudo randomly assigned as familiar or novel to each mouse to avoid object preference bias. The novel versus familiar side was randomly rotated between mice to account for side bias. Mouse behavior was recorded on video and traced with the software TopLightScan 2.0 (CleverSys, VA, USA). Mice with intact object recognition memory will show novelty preference and spend more time with the novel object rather than the familiar one. In this task, mice were exposed to treatment prior to the start of the task, thus assessing the impact of the treatments on memory formation.

### 2.8 Statistical analysis

Graphical representations and statistical analysis outputs were generated by GraphPad Prism (ver. 9.1.0, GraphPad Software, San Diego). All data were tested for normality using the Shapiro–Wilk test and passed normality. To analyze baseline locomotion, a two‐way ANOVA with sex and genotype as between factors was performed. For locomotor activity following drug challenge, a three‐way ANOVA was used with genotype and sex and time as between factors. For the first hour of OFT analysis and for body weight, a two‐way ANOVA with sex and genotype as between factors was performed. OFT analysis post drug‐exposure, EPM, latency to closed arm, and both novelty recognition tasks were analyzed by a three‐way ANOVA with sex, genotype, and treatment as between factors. In the case of a three‐way ANOVA, if an interaction with sex was detected, a separate analysis for males and females was performed followed by multiple comparison tests as recommended by the GraphPad Software. If there was no sex effect, female and male data were consolidated for further analysis. Outliers were removed by means of the ROUT test (*Q* = 5%). One mouse was removed from the locomotor data, one from the EPM data and one from the Y‐maze dataset due to being identified as a statistical outlier. Four mice were removed from the NORT dataset due to being identified as statistical outliers (all from different groups). In all cases, the significance level was set to *p* ≤ .05. Power analysis for 80% power using the three‐way ANOVA design requires an *n* of 8 for a medium effect (.75).

## 3 RESULTS

### 3.1 The effect of NMDAR antagonists on locomotion in WT and GluN2D‐KO mice

Mice were habituated for 1 h prior to drug challenge, then locomotor activity was assessed for 2 h post drug administration (Figure 2a). Locomotor activity assessed at baseline revealed a significant effect of time (*F*(8.5, 1284) = 126.2; *p* < .0001), genotype (*F*(1, 151) = 46.5; *p* < .0001) as well as time × genotype (*F*(11, 1661) = 1.9; *p* < .05) and time × sex × genotype interactions (*F*(11, 1661) = 2.8; *p* < .01) (Figure 2b). No main effect of sex was found. The main effect of time and genotype reflects a decrease in locomotor activity over time in all mice and a significantly lower baseline locomotor activity in GluN2DR KO mice when compared with WT controls, respectively.

Following drug challenge, three‐way ANOVA showed a significant main effect of drug (*F*(3, 103) = 7.713, *p* = .0001), a significant main effect of genotype (*F*(1, 103) = 32.62, *p* < .0001), and a significant drug × genotype interaction (*F*(3, 103) = 2.814, *p* = .04) (Figure 2c). There was no significant drug × sex × genotype interaction, so sexes were consolidated. Here, Sidak's multiple comparisons test showed significant differences between WT and KO groups following administration with R‐norket (*p* = .04) and PCP (*p* < .0001) with WT but not KO mice showing increased locomotor activity following drug administration but not S‐ket (*p* = .09) or saline (*p* = .49).

For the control saline group (Figure 2d), a RM three‐way ANOVA revealed a significant effect of time (*F*(4.012, 132.4) = 59.62; *p* < .0001) and time × genotype interaction (*F*(8, 264) = 4.011; *p* = .0002). As there was no effect of sex, female and male data were consolidated and Sidak's multiple comparisons showed significant genotype effects at 60 and 80 min, but not thereafter. This may reflect overall reduced activity from all mice in the task from 100 min onward.

Analysis of the R‐norket group (Figure 2e) from 60 min (time point of injection) onward revealed a significant main effect of time (*F*(2.702, 94.58) = 49.86; *p* < .0001) but no main effect of genotype or sex. The significant effect of time can reflect changes induced by the drug, given at 60 min. Here, while locomotion does appear to increase from 60 to 80 min, no significant difference was found at this time point, suggesting that R‐norket did not have a profound effect on locomotion in all mice. Significant reductions in locomotion were seen at 40 min post drug exposure (60 vs. 100, *p* = .0092) with all animals showing similar levels of reduced activity from 100 min onward. There was, a significant time × genotype interaction (*F*(8, 280) = 2.968; *p* = .0033), however post hoc comparisons revealed no significant genotype effects when assessed at each time bin. However, when data were averaged across the 2 h following exposure to R‐norket (Figure 2c), it appears that R‐norket increased locomotion to a greater extent in WT but not KO mice. Similar to the saline treated group, it appears that there is a genotype effect until approximately 100 min—after which all animals show reduced activity.

Analysis of the S‐ket group (Figure 2f) from 60 min (time point of injection) onward showed a significant main effect of time (*F*(4.116, 139.9) = 46.95; *p* < .0001) and a time × genotype interaction (*F*(8, 272) = 4.433; *p* = .0001). Post hoc comparisons for the main effect of time showed that similar to R‐norket, while there is a slight increase in locomotion 20 min post injection (at 80 min), this was not significant, indicating that the effects of the drug on locomotion were not profound. Locomotor activity then declines at 100 min in all mice and remains steady. However, when exploring the time × genotype interaction, Fisher's post hoc analysis showed that WT mice were significantly more active at 80 min (*p* = .008) and 100 min (*p* = .03) as compared to KO mice and this heightened activity declined thereafter reaching similar levels to KO, independent of sex. Thus, the increase in locomotor activity induced by S‐ketamine is significantly blunted in both male and female KO mice.

Analysis of the PCP group (Figure 2g) from 60 min onward showed a significant main effect of time (*F*(3.304, 118.9) = 25.59; *p* < .0001), genotype (*F*(1, 36) = 11.19; *p* = .0019), time × sex interaction (*F*(8, 288) = 3.794; *p* = .0003), time × genotype interaction (*F*(8, 288) = 4.539; *p* < .0001), and time × sex × genotype interaction (*F*(8, 288) = 2.690, *p* = .0072). Given the interaction with sex, female and male datasets were analyzed separately. Firstly, the main effect of time was explored in each sex, and we can see that the effect of drug is significant in males, with increased locomotion at 80 (*p* = .024) and 100 (*p* = .03) min post injection, while females show no significant differences in locomotion at the same time points. Genotype effects at each time bin were then explored in both females and males. Fisher's LSD test showed that WT female mice were significantly more active at 80 min (within 20 min post injection) as compared to KO mice (*p* = .027) and this heightened activity declined thereafter. In male mice, multiple comparison test showed that WT mice were significantly more active at 80 min (*p* = .008), 100 min (*p* = .02), and 120 min (*p* = .02) as compared to KO mice and this heightened activity declined thereafter reaching similar levels to KO mice by 140–180 min. Therefore, the increase in locomotor activity induced by PCP is significantly blunted in both male and female KO mice, although this effect is more prolonged in males.

We next assessed and compared the impact of each drug on ataxia, stereotypy, and catalepsy in a separate cohort of WT and KO male and female mice to determine if the locomotor activity described above may have been influenced by these behaviors. For ataxia (Figure 2h), a three‐way ANOVA showed a significant main effect of drug (*F*(2, 113) = 209.6; *p* < .0001), but no main effect of sex or genotype or any genotype × sex × drug interaction. Therefore, female and male data were consolidated and Tukey's post hoc analysis revealed that administration with R‐norket and S‐ket, but not PCP, produced ataxic behaviors in all mice, irrespective of genotype. Additionally, mice were significantly more ataxic following treatment with S‐ket compared to R‐norket (*p* < .0001).

For the stereotypy score (Figure 2i), three‐way ANOVA revealed a main effect of drug (*F*(2, 113) = 37.35; *p* < .0001) and sex (*F*(1, 113) = 3.992; *p* = .048) and a significant drug × sex interaction (*F*(2, 113) = 4.252; *p* = .017). Given the interaction with sex, female and male datasets were analyzed separately. A two‐way ANOVA for the female group revealed only a significant main effect of drug (*F*(2, 48) = 18.3; *p* < .0001) and the subsequent Tukey's post hoc analysis revealed that treatment with R‐norket (*p* = .0005) and S‐ket (*p* < .0001) both increased stereotypies when compared with PCP, which did not result in stereotypies in female mice. In male mice, a two‐way ANOVA showed only a main effect of drug. Subsequent Tukey's multiple comparison test showed that R‐norket and S‐ket produced stereotypic behavior and this was observed to a greater degree following treatment with S‐ket (*p* < .0001). However, PCP did not result in stereotypies. Furthermore, the increase in stereotypy induced by S‐ket was more pronounced in males than females (*p* = .0009).

Lastly when assessing cataleptic behavior (Figure 2j), a three‐way ANOVA revealed a significant main effect of drug (*F*(2, 113) = 27.35; *p* < .0001) but no other significant effects. Therefore, female and male data were consolidated and Tukey's multiple comparison test revealed that treatment with S‐ket compared to R‐norket (*p* < .0001) or PCP (*p* < .0001) resulted in catalepsy and to a similar degree in both genotypes. To summarize, treatment with 25 mg/kg of S‐ket causes ataxia, stereotypy, and catalepsy, treatment with 25 mg/kg R‐norket causes ataxia and stereotypy but to a lesser extent than S‐ket, and PCP at 3 mg/kg does not cause ataxia, stereotypy, or catalepsy. Therefore, the movement data shown in Figure 2c following PCP administration can be solely attributed to locomotion.

### 3.2 The effect of NMDAR antagonists on anxiety/fear‐related behavior in WT and GluN2D‐KO mice

**FIGURE 3:**
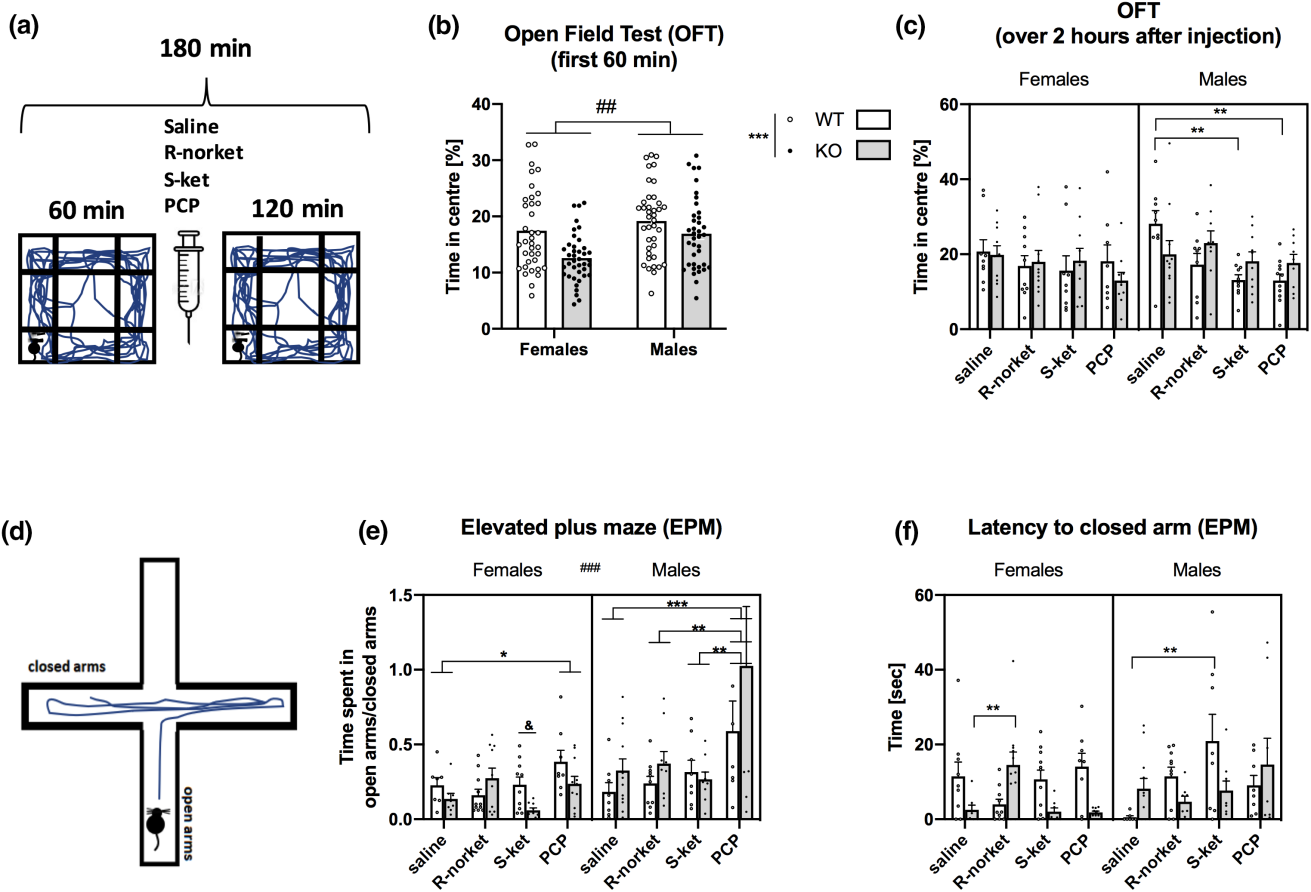
Anxiety/fear‐related behavior. (a) Open field test (OFT): The time spent in the center was recorded during the first hour (b) and during 2 h after injection with saline (*n* = 9 F WT, 10 F KO, 9 M WT, 11 M KO), R‐norket (*n* = 10 F WT, 11 F KO, 9 M WT, 9 M KO), S‐ket (9 F WT, 10 F KO, 10 M WT, 10 M KO), or PCP (8 F WT, 10 F KO, 11 M WT, 9 M KO) (c). (d) Elevated Plus Maze (EPM): The time spent in the open arms/closed arms over 5 min (e) and the latency to closed arm (f) was recorded. All data presented as mean ± SEM. When a two‐way ANOVA showed a significant interaction, *p*‐values were calculated with an ordinary two‐way ANOVA (b), Sidak's multiple comparison tests (c, e, f); *p < .05, **p < .01, ***p < .001, ^*p* < .0001, ^##^ *p* < .01 ‐main sex effect, ^&^ *p* < .01.

For the OFT, the time spent in the center was calculated—reduced time spent in the center is indicative of a more anxious phenotype (Figure 3a). During the first hour, before drug injection, all treatment groups were consolidated and a two‐way ANOVA revealed a significant main effect of genotype (*F*(1, 149) = 12.7; *p* < .001) and sex (*F*(1, 149) = 9.4; *p* < .01), but no genotype × sex interaction (Figure 3b). This showed that (a) KO mice spent less time in the center of the arena compared to WT controls indicative of anxiety‐like behavior and (b) female mice spent less time in the center compared to male mice, independent of genotype (Figure 3b). The average time spent in the center was calculated for each treatment group over 2 h after drug injection (Figure 3c). A three‐way ANOVA showed a significant main effect of drug (*F*(3, 139) = 4.133; *p* = .007), but no effect of sex or genotype and no interactions. Tukey's multiple comparisons for the main effect of drug showed significant differences between saline and S‐ket (*p* = .002) and saline and PCP (*p* = .0015) groups in male WT mice, with both drugs causing a reduction in the percentage of time spent in the center.

During the EPM test (Figure 3d), the time spent in open/closed arms negatively correlates with anxiety‐like phenotype. A three‐way ANOVA revealed a significant main effect of drug (*F*(3, 128) = 9.290; *p* < .0001), sex (*F*(1, 128) = 14.86; *p* = .0002), drug × sex interaction (*F*(3, 128) = 3.452; *p* = .02), and sex × genotype interaction (*F*(1, 128) = 5.258; *p* = .02). Data were then split by sex due to the above interactions. In female mice, a two‐way ANOVA showed a main effect of drug (*F*(3, 66) = 3.730; *p* = .01), a trend for an effect of genotype (*F*(1, 66) = 3.945; *p* = .051), and a drug × genotype interaction (*F*(3, 66) = 3.387; *p* = .02). Post hoc analysis for the main effect of drug showed a significant difference between the S‐ket and PCP groups (*p* = .01). Post hoc analysis for the drug × genotype interaction showed a trend for an effect of genotype in the S‐ket group (*p* = .08), with KO mice showing reduced time in the open arm/closed arm. Female KO mice in the saline and PCP groups also showed a nonsignificant reduction in time spent in the open/closed arm, but not the R‐norket group, suggesting the anxiogenic effect in the KO mice may be ameliorated only by R‐norket.

In male mice, there was a main effect of drug (*F*(3, 62) = 6.056; *p* = .001), but no effect of genotype and no drug × genotype interaction. Post hoc analysis for the main effect of drug showed that PCP had an anxiolytic effect in both WT and GluN2DR KO mice, but this effect was not seen with R‐norket or S‐ket (saline vs. PCP, *p* = .001; PCP vs. R‐norket, *p* = .005; PCP vs. S‐ket, *p* = .005).

Another aspect to consider, which can be regarded as initial short‐phasic fear response, is the time the mouse takes to hide in the closed arm after it was placed onto the open arm (Latency to closed arm, Figure 3f). The quicker it moves to the closed arm, it may be inferred the more anxious or fearful it is. Analysis showed no significant effect of sex or drug, but a trend for an effect of genotype (*F*(1, 128) = 3.881; *p* = .051) and a significant genotype × drug (*F*(3, 128) = 2.9; *p* = .03) and sex × genotype × drug interaction (*F*(3, 128) = 6.9; *p* = .0002). Further separate analysis for the females showed a main effect of genotype (*F*(1, 67) = 6.8; *p* = .01), no effect of drug, but a genotype × drug interaction (*F*(3, 67) = 8.7; *p* < .0001). The main effect of genotype showed that female KO mice were in general faster to hide in the closed arm as compared to female WT mice, again, indicating KO mice were more anxious than WT. To further explore the genotype × drug interaction, Sidak's comparisons test was performed and showed that R‐norket, but not S‐ket or PCP reversed this anxiogenic phenotype (*p* = .009) in KO females similar to the above findings that R‐norket reversed the reduced time spent in the open arm/closed arm. There were no significant effects of drug or genotype in the males, but a significant drug × genotype interaction (*F*(3, 61) = 3.2; *p* < .05). Further multiple comparison tests showed that only S‐ket had an anxiolytic effect in WT, but not in KO males (*p* = .006).

### 3.3 The effects of NMDAR antagonists on spatial recognition memory and novel object recognition memory in WT and GluN2D‐KO male and female mice

**FIGURE 4:**
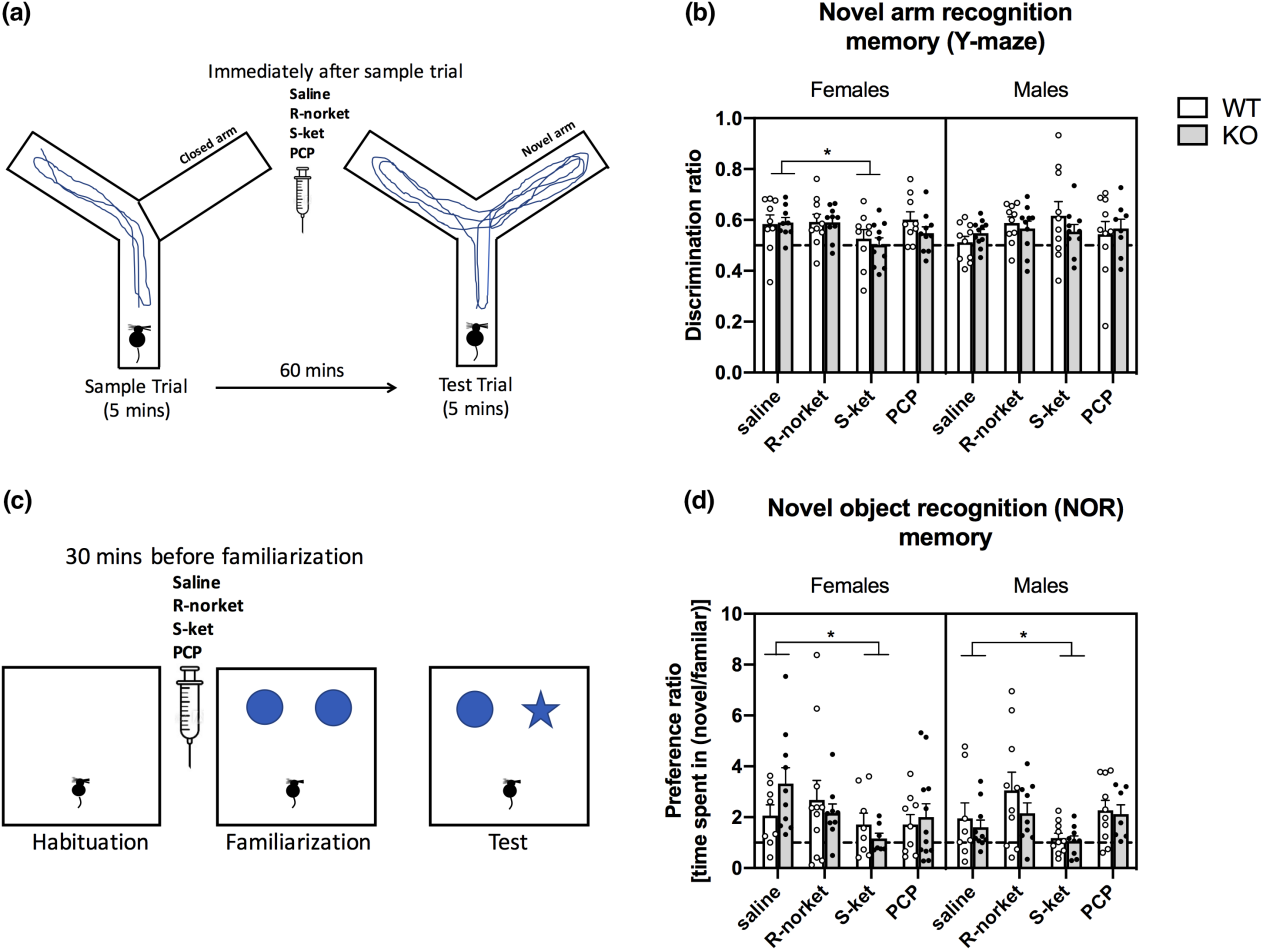
Novelty recognition memory. (a) Y‐maze task: After exploring two arms of the Y‐maze, mice were injected with either saline (*n* = 9 F WT, 9 F KO, 10 M WT, 11 M KO), R‐norket (*n* = 10 F WT, 11 F KO, 10 M WT, 10 M KO), S‐ket (9 F WT, 10 F KO, 10 M WT, 10 M KO), or PCP (9 F WT, 10 F KO, 10 M WT, 8 M KO). An hour later, the mice were returned to the Y‐maze and allowed to explore all three arms and their activity was recorded for 5 min; (b) S‐ket disrupts spatial memory in female but not male mice. (c) NOR task: After 2 days of habituation, mice were injected with either saline (*n* = 9 F WT, 10 F KO, 9 M WT, 11 M KO), R‐norket (*n* = 11 F WT, 9 F KO, 10 M WT, 9 M KO), S‐ket (9 F WT, 7 F KO, 10 M WT, 9 M KO), or PCP (9 F WT, 12 F KO, 10 M WT, 7 M KO) 30 min before being exposed to two novel objects for 10 min. An hour later, mice were exposed to one familiar object and one novel object and their activity was recorded for 5 min; (d) S‐ket disrupts novel object recognition in both male and female mice. All data presented as mean ± SEM. When a two‐way ANOVA showed a significant interaction, *p*‐values were calculated with the Dunnett's multiple comparisons test; *p < .05.

Y‐maze and NOR tasks were performed to assess novel arm (spatial) and novel object (episodic) recognition memory, respectively. Analysis of the Y‐maze did not reveal any main effect of sex, genotype, or drug, but a significant sex × drug interaction (*F*(3, 139) = 2.9; *p* < .05) (Figure 4a,b). Further separate analysis by sex showed that S‐ket, but not R‐norket or PCP, reduced the preference for the novel arm in female mice irrespective of genotype, which is indicative of impaired spatial short‐term memory 2‐way ANOVA, main treatment effect (*F*(3, 68) = 3.1; *p* < .05, Dunnett's multiple comparison test: saline vs. S‐ket *p = .038, Figure 4b). No effects of drugs or genotype and no interactions were found in the male groups. No group differences were observed in the percentage of time spent in the center, decision‐making phase, of the Y‐maze (data not shown).

Analysis for the NOR task revealed a significant main effect of treatment (*F*(3, 131) = 4.4; *p* < .01), but no other significant effects or interactions (Figure 4c,d). Dunnett's multiple comparison test showed that S‐ket, (*p = .024, Figure 4d) but not R‐norket or PCP, disrupted the preference for the novel object when compared to vehicle, independent of genotype or sex.

## 4 DISCUSSION

Here, we show, firstly, that the hyperlocomotion induced by the NMDAR antagonists, R‐norket, S‐ket, and PCP was blunted in both male and female GluN2D‐KO mice. We show that the baseline hypolocomotor phenotype in GluN2D‐KO mice, previously reported (Hagino et al., 2010; Ikeda et al., 1995; Miyamoto et al., 2002), is present in both sexes. We report that GluN2D‐KO mice exhibit anxiety‐like behaviors, in both the open field test and the elevated plus maze test, and this phenotype was more pronounced in female mice. The effect of NMDAR antagonist drugs on anxiety‐like behaviors was highly dependent on the task and sex of the mouse; in the OFT PCP, and S‐ket reduced the time spent in the center, which may suggest reduced exploratory behavior and increased fearful or anxious behavior. However, in the elevated plus maze task, R‐norket showed anxiolytic effects in females, while S‐ket showed anxiolytic effects in male WT but not KO mice. Furthermore, PCP also showed anxiolytic effects in this task that were independent of genotype. Finally, S‐ket was found to disrupt spatial recognition memory in females and novel object recognition memory in both sexes, independent of genotype.

As mentioned previously, several studies have reported that GluN2D‐KO mice exhibit decreased locomotor activity in novel environments when compared with WT mice. It has been suggested that this may be attributable to a reduction in motivation as GluN2D‐KO mice have been shown to display anhedonic‐ and depressive‐like behaviors (Salimando et al., 2020; Yamamoto et al., 2017). Furthermore, there is evidence of altered dopamine and serotonin metabolism in the frontal cortex, striatum, thalamus, and hippocampus of GluN2D‐KO mice which could explain the hypolocomotor behavior as well as the anhedonic state (Miyamoto et al., 2002). It is also well established that acute administration of NMDAR antagonists such as PCP and ketamine in mice results in hyperlocomotion, cognitive dysfunction, and deficits in social behaviors (Brigman et al., 2009). In this study, the hyperlocomotion induced by R‐norket, S‐ket, and PCP in WT mice was significantly reduced in GluN2D‐KO mice of both sexes. Thus, we provide here evidence that the GluN2D subunit may play a key role in hyperlocomotion induced by R‐norket, S‐ket, and PCP. PCP is considered to be a nonselective blocker of GluN2(A‐D)‐containing NMDA receptors but these results suggest that administration of PCP resulting in hyperlocomotion may be predominantly mediated by blocking the GluN2D, as previously shown (Sapkota et al., 2016; Yamamoto et al., 2013). We extend this by showing it is not sex specific. Although our findings of phenotypic differences in the effects of the different NMDAR antagonists is of interest given that ketamine and PCP have rarely been assessed together, a significant limitation of our study is that we only tested a single dose of each drug. Although this dose was chosen carefully based on previous studies (Fukumoto et al., 2017; Hagino et al., 2010), a full dose–response curve would be required to definitively confirm the differential effects of these drugs.

Only treatment with S‐ and R‐norket, but not PCP resulted in ataxia and stereotypies in this study and only S‐ket caused catalepsy. It is possible the lack of a PCP effect on ataxia and catalepsy behaviors could be dose related; however, previous studies report the absence of catalepsy or ataxia in mice following PCP treatment at 10 mg/kg (Koek & France, 2008). For these outcomes, there were no significant differences between the genotypes, so it is unlikely that NMDAR antagonist‐induced ataxia, catalepsy, or stereotypy is mediated by the GluN2D subunit. These data do, however, suggest the locomotor effects induced by R‐norket or S‐ket in this study may be influenced by stereotypy, ataxia, and catalepsy behaviors, even at the subanesthetic (25 mg/kg) dose that we applied—and may, in part, explain the modest effects that we found for S‐ket and R‐norket on locomotion. However, PCP had no effect on these behaviors but showed a very clear hyperlocomotion effect at the 3 mg/kg dose we administered. Given both ketamine and PCP have similar affinities for NMDA receptors, this effect may be due to the different doses applied, but also off‐target effects. For example, PCP has a greater potency as a D2 receptor activator than ketamine (Seeman et al., 2009) and D2 receptor activation is associated with hyperlocomotion.

In contrast to some previous studies, we report that GluN2D‐KO mice exhibit anxiety/fear‐related behavior during the OFT and EPM. Previously, Yamamoto et al. reported a normal level of anxiety‐like behavior in GluN2D‐KO mice using the marble‐burying test and novelty‐suppressed feeding test, while Miyamoto et al. reported reduced anxiety in GluN2D‐KO mice in the EPM and light–dark box test (Miyamoto et al., 2002; Yamamoto et al., 2017). The discrepancies in the findings might be due to the different tests used which might be sensitive to subtly different anxiety‐ or fear‐related behaviors. For example, recent studies have suggested that the marble‐burying test might more closely represent repetitive/compulsive behaviors than anxiety‐like behavior (Dixit et al., 2020; Thomas et al., 2009). Another contributing factor to the discrepancies in our findings compared to previous reports is that we assessed both sexes. Indeed, for the OFT, we found a main effect of genotype; however, this appears to be driven mainly by female mice and analysis of males alone may not have led to statistically significant results. Another recent study examining both male and female GluN2D‐KO mice also found increased anxiety‐ and depressive‐like behaviors in KO mice of both sexes which was associated with disruptions to the modulation of neural activity by GluN2D‐containing NMDARs in the bed nucleus of the stria terminalis, which is a complex structure known to modulate emotional states (Salimando et al., 2020). It is possible that sex differences in GluN2D levels and/or expression patterns might underlie some of the behavioral dimorphism observed in this study. However, there is very limited literature comparing GluN2D expression between sexes, largely due to a lack of knowledge of GluN2D expression in females. A study looking at relative expression of GluN2 variants found sex‐specific differences in the localization of the GluN2D subunit in the spinal dorsal horn of rats (Temi et al., 2021). Another study reported increased GluN2D expression in females diagnosed with major depressive disorder compared to controls, which was not seen in males (Gray et al., 2015).

Once again highlighting sex differences in anxiety‐ and fear‐related behaviors, female but not male KO mice show reduced time spent in the open arm of the elevated plus maze, and reduced latency to reach the closed arm. This suggests that the anxiety phenotype in the EPM is female specific, a finding unable to be assessed in the previous male only studies. We also show that R‐norket improves anxiety‐like behaviors in KO females—suggesting GluN2D is not involved in mediating the effects of R‐norket on this behavior in female mice. In line with our findings, a recent study reported that R‐ket attenuated anxiety‐related behaviors in an animal model of maternal immune activation which is commonly used to study neurodevelopmental disorders including schizophrenia (de Oliveira et al., 2023). This anxiolytic effect was linked to its antioxidant and anti‐inflammatory effects in the prefrontal cortex of the mice (de Oliveira et al., 2023). Thus, the anxiolytic effects of R‐norket observed in our study might be due to this anti‐inflammatory action rather than via GluN2D‐mediated signaling. S‐ket showed anxiolytic effects in male WT but not KO mice and PCP showed anxiolytic effects in both male and female WT and KO mice in the EPM task. Thus, we found almost opposing effects of PCP and the ketamine enantiomers in the EPM compared to the OFT tasks as PCP and S‐ket both reduced percentage time spent in the center of the OFT, suggestive of a fearful or anxiogenic effect. While reduced time spent in the center of the open field task can be seen as a sign of anxiety, the behavior may be confounded by locomotion, which we know at baseline is reduced in the KO mice, and is further altered by ketamine and PCP. We did account for this by calculating the percentage of time spent in the center, rather than raw time in the center; however, the elevated plus maze task may be a more reliable test of anxiety like behavior in this instance. The differing effects of R‐norket and S‐ket on these anxiety‐related behaviors might be explained by recent studies which report that S‐ketamine preferentially binds to and activates mu opioid receptors when compared with R‐ketamine (Bonaventura et al., 2021; Levinstein et al., 2023). Moreover, this interaction was linked to the antidepressant effects of ketamine as the administration of naltrexone, an opioid antagonist, blocked the antidepressant and anti‐suicidal effects of ketamine (Williams et al., 2018, 2019). This may explain the anxiolytic effects of S‐ket observed in male WT mice in our study.

As reported previously, we found that GluN2D‐KO mice had normal object recognition memory and this was disrupted in both genotypes and sexes by S‐Ket, but not R‐norket or PCP. One possible explanation for cognitive deficits following treatment with subanesthetic doses of NMDAR antagonists like S‐ket is the blockade of NMDAR specifically on parvalbumin+ GABAergic interneurons which play a key role in many cognitive processes. Blockade of the NMDARs on these interneurons would decrease GABA release resulting in the disinhibition of the pyramidal neurons (Widman & McMahon, 2018; Zhang, Yang, et al., 2021). This would in turn increase prefrontal glutamate release resulting in cortical hyperexcitability, disrupting the excitatory:inhibitory balance required for higher order cognitive function (Nakazawa et al., 2017; Nakazawa & Sapkota, 2020; Starc et al., 2017). Chronic treatment with S‐ket, but not R‐ket, was found to reduce PV immunoreactivity in the medial prefrontal cortex and hippocampus in mice (Yang et al., 2016). Thus, it is possible that administration of S‐ket causes disruption to their function in the prefrontal cortex and hippocampus resulting in cognitive deficits. Interestingly, recent studies report that R‐ketamine may in fact ameliorate cognitive deficits induced by maternal immune activation or treatment with PCP and this effect is not seen with S‐ketamine (Tan et al., 2020, 2022).

Ide et al. reported that R‐ket induced deficits in object recognition memory only in WT mice not in GluN2D‐KO mice, suggesting that the GluN2D subunit was important for cognitive impairment induced by this drug. Contrastingly, our results do not appear to show that PCP or R‐ket causes disruptions to object recognition memory or spatial recognition memory in any of the sexes or genotypes assessed. A possible explanation for this discrepancy is that while Ide et al. administered the drugs immediately after the familiarization phase (day 2) and then conducted the test phase 24 h later, we administered the drugs 30 min before the test phase of the NOR task. The protocol followed by Ide et al. would have been probing for the effect of NMDAR antagonists on memory consolidation whereas we were investigating the effect of the NMDAR antagonists on memory recall/retrieval (Lueptow, 2017). Thus, together these data suggest different roles for GluN2D subunit containing NMDARs in memory consolidation and retrieval.

A limitation of our study is that we used a sequential design for our behavioral testing with all mice performing the tests in the same order: locomotor test/OFT, Y‐maze, the EPM, and then the NOR task. All mice received only one type of NMDAR antagonist with a washout period of 48 h in between. Although this should be sufficient given a short elimination half‐life of approximately 13 min for ketamine and 46 min for PCP when administered i.p. in mice (Maxwell et al., 2006; Stone & Forney, 1978), there is a possibility of carryover effects due to the repeated administration of the compounds. Indeed, one study showed that subchronic ketamine (30 mg/kg once daily for 5 consecutive days) was sufficient to increase striatal dopamine synthesis and locomotor activity in mice (Kokkinou et al., 2021). Another study reported that subchronic PCP (1.5 mg/kg once daily for 4 days) impaired reversal learning in rats without affecting other nonspecific behaviors (Savolainen et al., 2021). Counterbalancing behavioral tests might account for some of the nonspecific effects of repeated exposure to drugs. Additionally, in this study, we did not test the role of GluN2D on the effects of the ketamine isomers on prepulse inhibition (PPI). PPI is a measure of sensorimotor gating consistently reported to be impaired in people with schizophrenia, and following treatment with NMDAR antagonists (Saletti et al., 2015; San‐Martin et al., 2020; Wu et al., 2018). S‐ket has been reported to disrupt PPI with greater potency than R‐ket in WT mice and rats (Halberstadt et al., 2016; Yang et al., 2015). Shelkar et al. reported a reduction in PPI in GluN2D‐KO mice compared to WT mice while others have reported that it is unaltered (Sapkota et al., 2016; Shelkar et al., 2019; Takeuchi et al., 2001). Thus, it is still unclear whether, and to what extent, the GluN2D subunit plays a role in PPI and should be further investigated.

In conclusion, our study confirms that the GluN2D subunit plays a key role in mediating the hyperlocomotor effects of NMDAR antagonists, but these effects are more pronounced in males than females. Both male and female GluN2D‐KO mice show an anxious phenotype, but this anxious phenotype is more pronounced in females. R‐norket and S‐ket showed anxiolytic properties that were dependent on sex and genotype, while PCP showed anxiolytic effects independent of sex and genotype. These data shed new light on sexually dimorphic responses to NMDAR antagonists and the role of the GluN2D subunit in mediating these effects.

## AUTHOR CONTRIBUTIONS

*Conceptualization*, S.S. and R.H.; *Methodology*, R.H., S.S., A.S., X.D., M.H. and N.J.; *Investigation*, C.V., A.S., and X.D.; *Supervision*, N.J., S.S., R.H.; *Project Administration*, S.S., R.H.; *Data Curation*, A.S., C.V., X.D., M.H., R.H.; *Formal Analysis*, C.V., A.S., X.D., and R.H.; *Writing – Original Draft*, C.V. and A.S.; *Writing – Review & Editing*, X.D., S.I., I.K., M.M., M.H., N.J., S.S., and R.H.; *Resources*, S.S., R.H., S.I., I.K., and M.M.

## FUNDING INFORMATION

This work was supported by a Society for Neurochemistry, Career Development Grant for senior author R.A. Hill as well as Departmental funding, Department of Psychiatry, Monash University. C.V. is supported by a Monash University scholarship. R.H. is supported by an NHMRC ideas grant (APP2000893).

## CONFLICT OF INTEREST STATEMENT

Kazutaka Ikeda has received speaker's fees from Araya Inc., Nippon Chemiphar Co., Ltd., EA Pharma Co., Ltd., Japan Tobacco, Inc., and Sumitomo Pharma Co., Ltd., consultancy honoraria from Nippon Chemiphar Co., Ltd. and Daiichi Sankyo Company Limited., and research grant from SBI Pharmaceuticals Co., Ltd. for a project unrelated to this research. Professor Suresh Sundram has received in the last 3 years speaker's fees, consultancy honoraria or research grants from Lundbeck, Otsuka, Seqirus, Janssen, Boehringer‐Ingelheim, Servier, and IHL unrelated to this study.

### PEER REVIEW

The peer review history for this article is available at https://www.webofscience.com/api/gateway/wos/peer‐review/10.1002/jnr.25257.

## Supporting information


**FIGURE S1** Body weight. GluN2D‐KO mice weighed less than WT controls independent of sex at (a) week 10 and (b) week 16. As expected, male mice were overall heavier than female mice at both time points. All data presented as mean ± SEM; ****p < .0001 main effect of genotype, ^####^ *p* < .0001 main effect of sex.

**TABLE S1** Three‐way ANOVA results for locomotor scoring assay.
**TABLE S2** Two‐way ANOVA results for locomotor scoring assay.
**TABLE S3** Results from Tukey's multiple comparisons test for Locomotor Scoring Assay.
**TABLE S4** Three‐way ANOVA results for locomotor test.
**TABLE S5** Two‐way ANOVA results for locomotor test.
**TABLE S6** Results from Sidak's multiple comparisons test for locomotor activity.
**TABLE S7** Results from Sidak's multiple comparisons test for locomotor activity.
**TABLE S8** Three‐way ANOVA results from open field test (OFT).
**TABLE S9** Two‐way ANOVA results from open field test (OFT).
**TABLE S10** Results from Sidak's multiple comparisons test for open field test (OFT).
**TABLE S11** Three‐way ANOVA results from elevated‐plus maze (EPM).
**TABLE S12** Two‐way ANOVA results from elevated‐plus maze (EPM).
**TABLE S13** Results from Sidak's multiple comparisons test for the elevated‐plus maze (EPM).
**TABLE S14** Results from Sidak's multiple comparisons test for the elevated‐plus maze (EPM).
**TABLE S15** Three‐way ANOVA results for the Y‐maze.
**TABLE S16** Two‐way ANOVA results for the Y‐maze.
**TABLE S17** Results from Dunnett's multiple comparisons test for the Y‐maze.
**TABLE S19** Results from Dunnett's multiple comparisons test for the Novel object recognition task (NORT).
**TABLE S18** Three‐way ANOVA results for the Novel object recognition task (NORT).

## Data Availability

Data can be made available upon request to the corresponding author.

## References

[jnr25257-bib-0001] Akazawa, C. , Shigemoto, R. , Bessho, Y. , Nakanishi, S. , & Mizuno, N. (1994). Differential expression of five N‐methyl‐d‐aspartate receptor subunit mRNAs in the cerebellum of developing and adult rats. Journal of Comparative Neurology, 347(1), 150–160. 10.1002/cne.903470112 7798379

[jnr25257-bib-0002] Akbarian, S. , Sucher, N. J. , Bradley, D. , Tafazzoli, A. , Trinh, D. , Hetrick, W. P. , Potkin, S. G. , Sandman, C. A. , Bunney, W. E., Jr. , & Jones, E. G. (1996). Selective alterations in gene expression for NMDA receptor subunits in prefrontal cortex of schizophrenics. Journal of Neuroscience, 16(1), 19–30. 8613785 10.1523/JNEUROSCI.16-01-00019.1996 PMC6578738

[jnr25257-bib-0003] Allen, R. M. , & Young, S. J. (1978). Phencyclidine‐induced psychosis. American Journal of Psychiatry, 135(9), 1081–1084. 696930 10.1176/ajp.135.9.1081

[jnr25257-bib-0004] Alsaad, H. A. , DeKorver, N. W. , Mao, Z. , Dravid, S. M. , Arikkath, J. , & Monaghan, D. T. (2019). In the telencephalon, GluN2C NMDA receptor subunit mRNA is predominately expressed in glial cells and GluN2D mRNA in interneurons. Neurochemical Research, 44, 61–77. 29651654 10.1007/s11064-018-2526-7 PMC6349034

[jnr25257-bib-0005] Antonoudiou, P. , Tan, Y. L. , Kontou, G. , Upton, A. L. , & Mann, E. O. (2020). Parvalbumin and somatostatin interneurons contribute to the generation of hippocampal gamma oscillations. Journal of Neuroscience, 40(40), 7668–7687. 32859716 10.1523/JNEUROSCI.0261-20.2020 PMC7531548

[jnr25257-bib-0006] Balu, D. T. (2016). The NMDA receptor and schizophrenia: From pathophysiology to treatment. In Advances in pharmacology (Vol. 76, pp. 351–382). Elsevier. 27288082 10.1016/bs.apha.2016.01.006 PMC5518924

[jnr25257-bib-0007] Bonaventura, J. , Lam, S. , Carlton, M. , Boehm, M. A. , Gomez, J. L. , Solís, O. , Sánchez‐Soto, M. , Morris, P. J. , Fredriksson, I. , Thomas, C. J. , Sibley, D. R. , Shaham, Y. , Zarate, C. A., Jr. , & Michaelides, M. (2021). Pharmacological and behavioral divergence of ketamine enantiomers: Implications for abuse liability. Molecular Psychiatry, 26(11), 6704–6722. 33859356 10.1038/s41380-021-01093-2 PMC8517038

[jnr25257-bib-0008] Brigman, J. L. , Ihne, J. , Saksida, L. M. , Bussey, T. , & Holmes, A. (2009). Effects of subchronic phencyclidine (PCP) treatment on social behaviors, and operant discrimination and reversal learning in C57BL/6J mice. Frontiers in Behavioral Neuroscience, 3, 2. 19255630 10.3389/neuro.08.002.2009 PMC2649201

[jnr25257-bib-0009] Cadinu, D. , Grayson, B. , Podda, G. , Harte, M. K. , Doostdar, N. , & Neill, J. C. (2018). NMDA receptor antagonist rodent models for cognition in schizophrenia and identification of novel drug treatments, an update. Neuropharmacology, 142, 41–62. 29196183 10.1016/j.neuropharm.2017.11.045

[jnr25257-bib-0010] Camargo, A. , Dalmagro, A. P. , Fraga, D. B. , Rosa, J. M. , Zeni, A. L. B. , Kaster, M. P. , & Rodrigues, A. L. S. (2021). Low doses of ketamine and guanosine abrogate corticosterone‐induced anxiety‐related behavior, but not disturbances in the hippocampal NLRP3 inflammasome pathway. Psychopharmacology, 238(9), 2555–2568. 10.1007/s00213-021-05879-8 34342672

[jnr25257-bib-0011] Canuso, C. M. , Singh, J. B. , Fedgchin, M. , Alphs, L. , Lane, R. , Lim, P. , Pinter, C. , Hough, D. , Sanacora, G. , Manji, H. , & Drevets, W. C. (2018). Efficacy and safety of intranasal esketamine for the rapid reduction of symptoms of depression and suicidality in patients at imminent risk for suicide: Results of a double‐blind, randomized, placebo‐controlled study. American Journal of Psychiatry, 175(7), 620–630. 29656663 10.1176/appi.ajp.2018.17060720

[jnr25257-bib-0012] Chang, L. , Zhang, K. , Pu, Y. , Qu, Y. , Wang, S.‐M. , Xiong, Z. , Ren, Q. , Dong, C. , Fujita, Y. , & Hashimoto, K. (2019). Comparison of antidepressant and side effects in mice after intranasal administration of (R, S)‐ketamine, (R)‐ketamine, and (S)‐ketamine. Pharmacology Biochemistry and Behavior, 181, 53–59. 31034852 10.1016/j.pbb.2019.04.008

[jnr25257-bib-0013] Chavez, C. , Gogos, A. , Jones, M. E. , & van den Buuse, M. (2009). Psychotropic drug‐induced locomotor hyperactivity and prepulse inhibition regulation in male and female aromatase knockout (ArKO) mice: Role of dopamine D1 and D2 receptors and dopamine transporters. Psychopharmacology, 206(2), 267–279. 10.1007/s00213-009-1604-6 19597801

[jnr25257-bib-0014] Chen, C.‐M. A. , Stanford, A. D. , Mao, X. , Abi‐Dargham, A. , Shungu, D. C. , Lisanby, S. H. , Schroeder, C. E. , & Kegeles, L. S. (2014). GABA level, gamma oscillation, and working memory performance in schizophrenia. Neuroimage: Clinical, 4, 531–539. 24749063 10.1016/j.nicl.2014.03.007 PMC3989525

[jnr25257-bib-0015] Cheng, W.‐J. , Chen, C.‐H. , Chen, C.‐K. , Huang, M.‐C. , Pietrzak, R. H. , Krystal, J. H. , & Xu, K. (2018). Similar psychotic and cognitive profile between ketamine dependence with persistent psychosis and schizophrenia. Schizophrenia Research, 199, 313–318. 29510925 10.1016/j.schres.2018.02.049

[jnr25257-bib-0016] Chung, D. W. , Geramita, M. A. , & Lewis, D. A. (2022). Synaptic variability and cortical gamma oscillation power in schizophrenia. American Journal of Psychiatry, 179(4), 277–287. 35360919 10.1176/appi.ajp.2021.21080798 PMC9580070

[jnr25257-bib-0017] Cohen, S. M. , Tsien, R. W. , Goff, D. C. , & Halassa, M. M. (2015). The impact of NMDA receptor hypofunction on GABAergic neurons in the pathophysiology of schizophrenia. Schizophrenia Research, 167(1–3), 98–107. 25583246 10.1016/j.schres.2014.12.026 PMC4724170

[jnr25257-bib-0018] Daly, E. J. , Turkoz, I. , Salvadore, G. , Fedgchin, M. , Ionescu, D. F. , Starr, H. L. , Borentain, S. , Trivedi, M. H. , Thase, M. E. , & Singh, J. B. (2021). The effect of esketamine in patients with treatment‐resistant depression with and without comorbid anxiety symptoms or disorder. Depression and Anxiety, 38(11), 1120–1130. 34293233 10.1002/da.23193 PMC9291524

[jnr25257-bib-0019] de Oliveira, E. G. , de Lima, D. A. , da Silva Júnior, J. C. , de Souza Barbosa, M. V. , de Andrade Silva, S. C. , de Santana, J. H. , dos Santos Junior, O. H. , Lira, E. C. , Lagranha, C. J. , & Duarte, F. S. (2023). (R)‐ketamine attenuates neurodevelopmental disease‐related phenotypes in a mouse model of maternal immune activation. European Archives of Psychiatry and Clinical Neuroscience, 273, 1–12. 37249625 10.1007/s00406-023-01629-3

[jnr25257-bib-0020] Dienel, S. J. , & Lewis, D. A. (2019). Alterations in cortical interneurons and cognitive function in schizophrenia. Neurobiology of Disease, 131, 104208. 29936230 10.1016/j.nbd.2018.06.020 PMC6309598

[jnr25257-bib-0021] Dixit, P. V. , Sahu, R. , & Mishra, D. K. (2020). Marble‐burying behavior test as a murine model of compulsive‐like behavior. Journal of Pharmacological and Toxicological Methods, 102, 106676. 31954839 10.1016/j.vascn.2020.106676

[jnr25257-bib-0022] Domino, E. F. , & Warner, D. S. (2010). Taming the ketamine tiger. Journal of the American Society of Anesthesiologists, 113(3), 678–684. 10.1097/ALN.0b013e3181ed09a2 20693870

[jnr25257-bib-0023] Driesen, N. R. , McCarthy, G. , Bhagwagar, Z. , Bloch, M. H. , Calhoun, V. D. , D'Souza, D. C. , Gueorguieva, R. , He, G. , Leung, H. C. , Ramani, R. , Anticevic, A. , Suckow, R. F. , Morgan, P. T. , & Krystal, J. H. (2013). The impact of NMDA receptor blockade on human working memory‐related prefrontal function and connectivity. Neuropsychopharmacology, 38(13), 2613–2622. 23856634 10.1038/npp.2013.170 PMC3828532

[jnr25257-bib-0024] Ebert, B. , Mikkelsen, S. , Thorkildsen, C. , & Borgbjerg, F. M. (1997). Norketamine, the main metabolite of ketamine, is a non‐competitive NMDA receptor antagonist in the rat cortex and spinal cord. European Journal of Pharmacology, 333(1), 99–104. 9311667 10.1016/s0014-2999(97)01116-3

[jnr25257-bib-0025] Engelhardt, J. V. , Bocklisch, C. , Tönges, L. , Herb, A. , Mishina, M. , & Monyer, H. (2015). GluN2D‐containing NMDA receptors‐mediate synaptic currents in hippocampal interneurons and pyramidal cells in juvenile mice. Frontiers in Cellular Neuroscience, 9, 95. 25859181 10.3389/fncel.2015.00095 PMC4373385

[jnr25257-bib-0026] Enwright, J. F., III , Huo, Z. , Arion, D. , Corradi, J. P. , Tseng, G. , & Lewis, D. A. (2018). Transcriptome alterations of prefrontal cortical parvalbumin neurons in schizophrenia. Molecular Psychiatry, 23(7), 1606–1613. 29112193 10.1038/mp.2017.216 PMC5938166

[jnr25257-bib-0027] Enwright, J. F. , Sanapala, S. , Foglio, A. , Berry, R. , Fish, K. N. , & Lewis, D. A. (2016). Reduced labeling of parvalbumin neurons and perineuronal nets in the dorsolateral prefrontal cortex of subjects with schizophrenia. Neuropsychopharmacology, 41(9), 2206–2214. 26868058 10.1038/npp.2016.24 PMC4946056

[jnr25257-bib-0028] Fitzgerald, P. J. , Hale, P. J. , Ghimire, A. , & Watson, B. O. (2021). Repurposing cholinesterase inhibitors as antidepressants? Dose and stress‐sensitivity may be critical to opening possibilities. Frontiers in Behavioral Neuroscience, 14, 620119. 33519395 10.3389/fnbeh.2020.620119 PMC7840590

[jnr25257-bib-0029] Fraga, D. B. , Olescowicz, G. , Moretti, M. , Siteneski, A. , Tavares, M. K. , Azevedo, D. , Colla, A. R. S. , & Rodrigues, A. L. S. (2018). Anxiolytic effects of ascorbic acid and ketamine in mice. Journal of Psychiatric Research, 100, 16–23. 10.1016/j.jpsychires.2018.02.006 29475017

[jnr25257-bib-0030] Fukumoto, K. , Toki, H. , Iijima, M. , Hashihayata, T. , Yamaguchi, J.‐I. , Hashimoto, K. , & Chaki, S. (2017). Antidepressant potential of (R)‐ketamine in rodent models: Comparison with (S)‐ketamine. Journal of Pharmacology and Experimental Therapeutics, 361(1), 9–16. 28115553 10.1124/jpet.116.239228

[jnr25257-bib-0031] Garst‐Orozco, J. , Malik, R. , Lanz, T. A. , Weber, M. L. , Xi, H. , Arion, D. , Enwright, J. F., 3rd , Lewis, D. A. , O'Donnell, P. , Sohal, V. S. , & Buhl, D. L. (2020). GluN2D‐mediated excitatory drive onto medial prefrontal cortical PV+ fast‐spiking inhibitory interneurons. PLoS ONE, 15(6), e0233895. 32497062 10.1371/journal.pone.0233895 PMC7272025

[jnr25257-bib-0032] Gigg, J. , McEwan, F. , Smausz, R. , Neill, J. , & Harte, M. K. (2020). Synaptic biomarker reduction and impaired cognition in the sub‐chronic PCP mouse model for schizophrenia. Journal of Psychopharmacology, 34(1), 115–124. 31580184 10.1177/0269881119874446

[jnr25257-bib-0033] Gonzalez‐Burgos, G. , Cho, R. Y. , & Lewis, D. A. (2015). Alterations in cortical network oscillations and parvalbumin neurons in schizophrenia. Biological Psychiatry, 77(12), 1031–1040. 25863358 10.1016/j.biopsych.2015.03.010 PMC4444373

[jnr25257-bib-0034] Gray, A. , Hyde, T. , Deep‐Soboslay, A. , Kleinman, J. , & Sodhi, M. (2015). Sex differences in glutamate receptor gene expression in major depression and suicide. Molecular Psychiatry, 20(9), 1057–1068. 26169973 10.1038/mp.2015.91

[jnr25257-bib-0035] Grech, A. M. , Du, X. , Murray, S. S. , Xiao, J. , & Hill, R. A. (2019). Sex‐specific spatial memory deficits in mice with a conditional TrkB deletion on parvalbumin interneurons. Behavioural Brain Research, 372, 111984. 10.1016/j.bbr.2019.111984 31150746

[jnr25257-bib-0036] Hagino, Y. , Kasai, S. , Han, W. , Yamamoto, H. , Nabeshima, T. , Mishina, M. , & Ikeda, K. (2010). Essential role of NMDA receptor channel ε4 subunit (GluN2D) in the effects of phencyclidine, but not methamphetamine. PLoS ONE, 5(10), e13722. 21060893 10.1371/journal.pone.0013722 PMC2965660

[jnr25257-bib-0037] Halberstadt, A. L. , Slepak, N. , Hyun, J. , Buell, M. R. , & Powell, S. B. (2016). The novel ketamine analog methoxetamine produces dissociative‐like behavioral effects in rodents. Psychopharmacology, 233, 1215–1225. 26758284 10.1007/s00213-016-4203-3 PMC5403250

[jnr25257-bib-0038] Hansen, K. B. , Yi, F. , Perszyk, R. E. , Menniti, F. S. , & Traynelis, S. F. (2017). NMDA receptors in the central nervous system. NMDA Receptors: Methods and Protocols, 1677, 1–80. 10.1007/978-1-4939-7321-7_1 PMC7325486 28986865

[jnr25257-bib-0039] Hanson, E. , Armbruster, M. , Lau, L. A. , Sommer, M. E. , Klaft, Z.‐J. , Swanger, S. A. , Traynelis, S. F. , Moss, S. J. , Noubary, F. , Chadchankar, J. , & Dulla, C. G. (2019). Tonic activation of GluN2C/GluN2D‐containing NMDA receptors by ambient glutamate facilitates cortical interneuron maturation. Journal of Neuroscience, 39(19), 3611–3626. 30846615 10.1523/JNEUROSCI.1392-18.2019 PMC6510335

[jnr25257-bib-0040] Henson, M. A. , Roberts, A. C. , Salimi, K. , Vadlamudi, S. , Hamer, R. M. , Gilmore, J. H. , Jarskog, L. F. , & Philpot, B. D. (2008). Developmental regulation of the NMDA receptor subunits, NR3A and NR1, in human prefrontal cortex. Cerebral Cortex, 18(11), 2560–2573. 18296432 10.1093/cercor/bhn017 PMC2733318

[jnr25257-bib-0041] Hou, L. , Miao, J. , Meng, H. , Liu, X. , Wang, D. , Tan, Y. , & Li, C. (2022). Sirtuin type 1 mediates the antidepressant effect of S‐ketamine in a chronic unpredictable stress model. Frontiers in Psychiatry, 13, 855810. 10.3389/fpsyt.2022.855810 35664490 PMC9160425

[jnr25257-bib-0042] Hudson, M. R. , Sokolenko, E. , O'Brien, T. J. , & Jones, N. C. (2020). NMDA receptors on parvalbumin‐positive interneurons and pyramidal neurons both contribute to MK‐801 induced gamma oscillatory disturbances: Complex relationships with behaviour. Neurobiology of Disease, 134, 104625. 31786371 10.1016/j.nbd.2019.104625

[jnr25257-bib-0043] Ide, S. , Ikekubo, Y. , Mishina, M. , Hashimoto, K. , & Ikeda, K. (2019). Cognitive impairment that is induced by (R)‐ketamine is abolished in NMDA GluN2D receptor subunit knockout mice. International Journal of Neuropsychopharmacology, 22(7), 449–452. 31135879 10.1093/ijnp/pyz025 PMC6600477

[jnr25257-bib-0044] Ikeda, K. , Araki, K. , Takayama, C. , Inoue, Y. , Yagi, T. , Aizawa, S. , & Mishina, M. (1995). Reduced spontaneous activity of mice defective in the ε4 subunit of the NMDA receptor channel. Molecular Brain Research, 33(1), 61–71. 8774946 10.1016/0169-328x(95)00107-4

[jnr25257-bib-0045] Jelen, L. A. , Young, A. H. , & Stone, J. M. (2021). Ketamine: A tale of two enantiomers. Journal of Psychopharmacology, 35(2), 109–123. 33155503 10.1177/0269881120959644 PMC7859674

[jnr25257-bib-0046] Kaar, S. J. , Angelescu, I. , Marques, T. R. , & Howes, O. D. (2019). Pre‐frontal parvalbumin interneurons in schizophrenia: A meta‐analysis of post‐mortem studies. Journal of Neural Transmission, 126, 1637–1651. 31529297 10.1007/s00702-019-02080-2 PMC6856257

[jnr25257-bib-0047] Karakas, E. , & Furukawa, H. (2014). Crystal structure of a heterotetrameric NMDA receptor ion channel. Science, 344(6187), 992–997. 24876489 10.1126/science.1251915 PMC4113085

[jnr25257-bib-0048] Klug, M. , Hill, R. A. , Choy, K. H. C. , Kyrios, M. , Hannan, A. J. , & van den Buuse, M. (2012). Long‐term behavioral and NMDA receptor effects of young‐adult corticosterone treatment in BDNF heterozygous mice. Neurobiology of Disease, 46(3), 722–731. 10.1016/j.nbd.2012.03.015 22426399

[jnr25257-bib-0049] Koek, W. , & France, C. P. (2008). Cataleptic effects of γ‐hydroxybutyrate (GHB) and baclofen in mice: Mediation by GABAB receptors, but differential enhancement by N‐methyl‐d‐aspartate (NMDA) receptor antagonists. Psychopharmacology, 199(2), 191–198. 18446324 10.1007/s00213-008-1160-5 PMC3470870

[jnr25257-bib-0050] Kokkinou, M. , Irvine, E. E. , Bonsall, D. R. , Natesan, S. , Wells, L. A. , Smith, M. , Glegola, J. , Paul, E. J. , Tossell, K. , Veronese, M. , Khadayate, S. , Dedic, N. , Hopkins, S. C. , Ungless, M. A. , Withers, D. J. , & Howes, O. D. (2021). Reproducing the dopamine pathophysiology of schizophrenia and approaches to ameliorate it: A translational imaging study with ketamine. Molecular Psychiatry, 26(6), 2562–2576. 32382134 10.1038/s41380-020-0740-6 PMC8440182

[jnr25257-bib-0051] Krystal, J. H. , Karper, L. P. , Seibyl, J. P. , Freeman, G. K. , Delaney, R. , Bremner, J. D. , Heninger, G. R. , Bowers, M. B., Jr. , & Charney, D. S. (1994). Subanesthetic effects of the noncompetitive NMDA antagonist, ketamine, in humans. Psychotomimetic, perceptual, cognitive, and neuroendocrine responses. Archives of General Psychiatry, 51(3), 199–214. 10.1001/archpsyc.1994.03950030035004 8122957

[jnr25257-bib-0052] Lahti, A. C. , Koffel, B. , LaPorte, D. , & Tamminga, C. A. (1995). Subanesthetic doses of ketamine stimulate psychosis in schizophrenia. Neuropsychopharmacology, 13(1), 9–19. 10.1016/0893-133X(94)00131-I 8526975

[jnr25257-bib-0053] Leal, G. C. , Bandeira, I. D. , Correia‐Melo, F. S. , Telles, M. , Mello, R. P. , Vieira, F. , Lima, C. S. , Jesus‐Nunes, A. P. , Guerreiro‐Costa, L. N. F. , Marback, R. F. , Caliman‐Fontes, A. T. , Marques, B. L. S. , Bezerra, M. L. O. , Dias‐Neto, A. L. , Silva, S. S. , Sampaio, A. S. , Sanacora, G. , Turecki, G. , Loo, C. , … Quarantini, L. C. (2021). Intravenous arketamine for treatment‐resistant depression: Open‐label pilot study. European Archives of Psychiatry and Clinical Neuroscience, 271, 577–582. 32078034 10.1007/s00406-020-01110-5

[jnr25257-bib-0054] Levinstein, M. R. , Carlton, M. L. , Di Ianni, T. , Ventriglia, E. N. , Rizzo, A. , Gomez, J. L. , Budinich, R. C. , Shaham, Y. , Airan, R. D. , Zarate, C. A., Jr. , Bonaventura, J. , & Michaelides, M. (2023). Mu opioid receptor activation mediates (S)‐ketamine reinforcement in rats: Implications for abuse liability. Biological Psychiatry, 93(12), 1118–1126. 36841701 10.1016/j.biopsych.2022.12.019 PMC11947972

[jnr25257-bib-0055] Liang, H. J. , Lau, C. G. , Tang, K. L. A. , Chan, F. , Ungvari, G. S. , & Tang, W. K. (2014). Are sexes affected differently by ketamine? An exploratory study in ketamine users. Substance use & Misuse, 49(4), 395–404. 24106975 10.3109/10826084.2013.841248

[jnr25257-bib-0056] Luby, E. D. , Cohen, B. D. , Rosenbaum, G. , Gottlieb, J. S. , & Kelley, R. (1959). Study of a new schizophrenomimetic drug—Sernyl. AMA Archives of Neurology & Psychiatry, 81(3), 363–369. 13626287 10.1001/archneurpsyc.1959.02340150095011

[jnr25257-bib-0057] Lueptow, L. M. (2017). Novel object recognition test for the investigation of learning and memory in mice. Journal of Visualized Experiments, 126, e55718. 10.3791/55718 PMC5614391 28892027

[jnr25257-bib-0058] Maxwell, C. R. , Ehrlichman, R. S. , Liang, Y. , Trief, D. , Kanes, S. J. , Karp, J. , & Siegel, S. J. (2006). Ketamine produces lasting disruptions in encoding of sensory stimuli. Journal of Pharmacology and Experimental Therapeutics, 316(1), 315–324. 16192313 10.1124/jpet.105.091199

[jnr25257-bib-0059] Mendrek, A. , & Mancini‐Marie, A. (2016). Sex/gender differences in the brain and cognition in schizophrenia. Neuroscience and Biobehavioral Reviews, 67, 57–78. 10.1016/j.neubiorev.2015.10.013 26743859

[jnr25257-bib-0060] Miyamoto, Y. , Yamada, K. , Noda, Y. , Mori, H. , Mishina, M. , & Nabeshima, T. (2002). Lower sensitivity to stress and altered monoaminergic neuronal function in mice lacking the NMDA receptor ε4 subunit. Journal of Neuroscience, 22(6), 2335–2342. 11896172 10.1523/JNEUROSCI.22-06-02335.2002 PMC6758257

[jnr25257-bib-0061] Monyer, H. , Burnashev, N. , Laurie, D. J. , Sakmann, B. , & Seeburg, P. H. (1994). Developmental and regional expression in the rat brain and functional properties of four NMDA receptors. Neuron, 12(3), 529–540. 10.1016/0896-6273(94)90210-0 7512349

[jnr25257-bib-0062] Nakazawa, K. , Jeevakumar, V. , & Nakao, K. (2017). Spatial and temporal boundaries of NMDA receptor hypofunction leading to schizophrenia. NPJ Schizophrenia, 3(1), 7. 28560253 10.1038/s41537-016-0003-3 PMC5441533

[jnr25257-bib-0063] Nakazawa, K. , & Sapkota, K. (2020). The origin of NMDA receptor hypofunction in schizophrenia. Pharmacology & Therapeutics, 205, 107426. 31629007 10.1016/j.pharmthera.2019.107426 PMC6981256

[jnr25257-bib-0064] Neill, J. C. , Harte, M. K. , Haddad, P. M. , Lydall, E. S. , & Dwyer, D. M. (2014). Acute and chronic effects of NMDA receptor antagonists in rodents, relevance to negative symptoms of schizophrenia: A translational link to humans. European Neuropsychopharmacology, 24(5), 822–835. 24287012 10.1016/j.euroneuro.2013.09.011

[jnr25257-bib-0065] Paoletti, P. , Bellone, C. , & Zhou, Q. (2013). NMDA receptor subunit diversity: Impact on receptor properties, synaptic plasticity and disease. Nature Reviews Neuroscience, 14(6), 383–400. 23686171 10.1038/nrn3504

[jnr25257-bib-0066] Perszyk, R. E. , DiRaddo, J. O. , Strong, K. L. , Low, C. M. , Ogden, K. K. , Khatri, A. , Vargish, G. A. , Pelkey, K. A. , Tricoire, L. , Liotta, D. C. , Smith, Y. , McBain, C. J. , & Traynelis, S. F. (2016). GluN2D‐containing N‐methyl‐d‐aspartate receptors mediate synaptic transmission in hippocampal interneurons and regulate interneuron activity. Molecular Pharmacology, 90(6), 689–702. 10.1124/mol.116.105130 27625038 PMC5118640

[jnr25257-bib-0067] Plataki, M. E. , Diskos, K. , Sougklakos, C. , Velissariou, M. , Georgilis, A. , Stavroulaki, V. , & Sidiropoulou, K. (2021). Effect of neonatal treatment with the NMDA receptor antagonist, MK‐801, during different temporal windows of postnatal period in adult prefrontal cortical and hippocampal function. Frontiers in Behavioral Neuroscience, 15, 689193. 34177484 10.3389/fnbeh.2021.689193 PMC8230549

[jnr25257-bib-0068] Rafało‐Ulińska, A. , & Pałucha‐Poniewiera, A. (2022). The effectiveness of (R)‐ketamine and its mechanism of action differ from those of (S)‐ketamine in a chronic unpredictable mild stress model of depression in C57BL/6J mice. Behavioural Brain Research, 418, 113633. 34673124 10.1016/j.bbr.2021.113633

[jnr25257-bib-0069] Ribeiro, M. , Brigas, H. C. , Temido‐Ferreira, M. , Pousinha, P. A. , Regen, T. , Santa, C. , Coelho, J. E. , Marques‐Morgado, I. , Valente, C. A. , Omenetti, S. , Stockinger, B. , Waisman, A. , Manadas, B. , Lopes, L. V. , Silva‐Santos, B. , & Ribot, J. C. (2019). Meningeal γδ T cell–derived IL‐17 controls synaptic plasticity and short‐term memory. Science Immunology, 4(40), eaay5199. 10.1126/sciimmunol.aay5199 31604844 PMC6894940

[jnr25257-bib-0070] Sahin, C. , Doostdar, N. , & Neill, J. C. (2016). Towards the development of improved tests for negative symptoms of schizophrenia in a validated animal model. Behavioural Brain Research, 312, 93–101. 27312268 10.1016/j.bbr.2016.06.021

[jnr25257-bib-0071] Saletti, P. G. , Maior, R. S. , Hori, E. , Nishijo, H. , & Tomaz, C. (2015). Sensorimotor gating impairments induced by MK‐801 treatment may be reduced by tolerance effect and by familiarization in monkeys. Frontiers in Pharmacology, 6, 204. 26441660 10.3389/fphar.2015.00204 PMC4585034

[jnr25257-bib-0072] Salimando, G. J. , Hyun, M. , Boyt, K. M. , & Winder, D. G. (2020). BNST GluN2D‐containing NMDA receptors influence anxiety‐and depressive‐like behaviors and modulate cell‐specific excitatory/inhibitory synaptic balance. Journal of Neuroscience, 40(20), 3949–3968. 32277042 10.1523/JNEUROSCI.0270-20.2020 PMC7219300

[jnr25257-bib-0073] San‐Martin, R. , Castro, L. A. , Menezes, P. R. , Fraga, F. J. , Simões, P. W. , & Salum, C. (2020). Meta‐analysis of sensorimotor gating deficits in patients with schizophrenia evaluated by prepulse inhibition test. Schizophrenia Bulletin, 46(6), 1482–1497. 32506125 10.1093/schbul/sbaa059 PMC8061122

[jnr25257-bib-0074] Sapkota, K. , Mao, Z. , Synowicki, P. , Lieber, D. , Liu, M. , Ikezu, T. , Gautam, V. , & Monaghan, D. T. (2016). GluN2D N‐methyl‐d‐aspartate receptor subunit contribution to the stimulation of brain activity and gamma oscillations by ketamine: Implications for schizophrenia. Journal of Pharmacology and Experimental Therapeutics, 356(3), 702–711. 10.1124/jpet.115.230391 26675679 PMC4767398

[jnr25257-bib-0075] Savolainen, K. , Ihalainen, J. , Hämäläinen, E. , Tanila, H. , & Forsberg, M. M. (2021). Phencyclidine‐induced cognitive impairments in repeated touchscreen visual reversal learning tests in rats. Behavioural Brain Research, 404, 113057. 33316322 10.1016/j.bbr.2020.113057

[jnr25257-bib-0076] Scotton, E. , Antqueviezc, B. , Vasconcelos, M. , Dalpiaz, G. , Géa, L. P. , Goularte, J. F. , Colombo, R. , & Rosa, A. R. (2022). Is (R)‐ketamine a potential therapeutic agent for treatment‐resistant depression with less detrimental side effects? A review of molecular mechanisms underlying ketamine and its enantiomers. Biochemical Pharmacology, 198, 114963. 35182519 10.1016/j.bcp.2022.114963

[jnr25257-bib-0077] Seeman, P. , Guan, H. C. , & Hirbec, H. (2009). Dopamine D2High receptors stimulated by phencyclidines, lysergic acid diethylamide, salvinorin a, and modafinil. Synapse, 63(8), 698–704. 10.1002/syn.20647 19391150

[jnr25257-bib-0078] Shelkar, G. P. , Pavuluri, R. , Gandhi, P. J. , Ravikrishnan, A. , Gawande, D. Y. , Liu, J. , Stairs, D. J. , Ugale, R. R. , & Dravid, S. M. (2019). Differential effect of NMDA receptor GluN2C and GluN2D subunit ablation on behavior and channel blocker‐induced schizophrenia phenotypes. Scientific Reports, 9(1), 7572. 31110197 10.1038/s41598-019-43957-2 PMC6527682

[jnr25257-bib-0079] Singh, J. B. , Fedgchin, M. , Daly, E. , Xi, L. , Melman, C. , De Bruecker, G. , Tadic, A. , Sienaert, P. , Wiegand, F. , Manji, H. , Drevets, W. C. , & van Nueten, L. (2016). Intravenous esketamine in adult treatment‐resistant depression: A double‐blind, double‐randomization, placebo‐controlled study. Biological Psychiatry, 80(6), 424–431. 26707087 10.1016/j.biopsych.2015.10.018

[jnr25257-bib-0080] Starc, M. , Murray, J. D. , Santamauro, N. , Savic, A. , Diehl, C. , Cho, Y. T. , Srihari, V. , Morgan, P. T. , Krystal, J. H. , Wang, X. J. , Repovs, G. , & Anticevic, A. (2017). Schizophrenia is associated with a pattern of spatial working memory deficits consistent with cortical disinhibition. Schizophrenia Research, 181, 107–116. 27745755 10.1016/j.schres.2016.10.011 PMC5901719

[jnr25257-bib-0081] Stone, C. J. , & Forney, R. B. (1978). The effects of cannabidiol or delta‐9‐tetrahydrocannabinol on phencyclidine‐induced activity in mice. Toxicology Letters, 1(5–6), 331–335.

[jnr25257-bib-0082] Suryavanshi, P. S. , Ugale, R. , Yilmazer‐Hanke, D. , Stairs, D. J. , & Dravid, S. (2014). GluN2C/GluN2D subunit‐selective NMDA receptor potentiator CIQ reverses MK‐801‐induced impairment in prepulse inhibition and working memory in Y‐maze test in mice. British Journal of Pharmacology, 171(3), 799–809. 24236947 10.1111/bph.12518 PMC3969090

[jnr25257-bib-0083] Takeuchi, T. , Kiyama, Y. , Nakamura, K. , Tsujita, M. , Matsuda, I. , Mori, H. , Munemoto, Y. , Kuriyama, H. , Natsume, R. , Sakimura, K. , & Mishina, M. (2001). Roles of the glutamate receptor ε2 and δ2 subunits in the potentiation and prepulse inhibition of the acoustic startle reflex. European Journal of Neuroscience, 14(1), 153–160. 11488959 10.1046/j.0953-816x.2001.01620.x

[jnr25257-bib-0084] Tan, Y. , Fujita, Y. , Pu, Y. , Chang, L. , Qu, Y. , Wang, X. , & Hashimoto, K. (2022). Repeated intermittent administration of (R)‐ketamine during juvenile and adolescent stages prevents schizophrenia‐relevant phenotypes in adult offspring after maternal immune activation: A role of TrkB signaling. European Archives of Psychiatry and Clinical Neuroscience, 272, 1–9. 10.1007/s00406-021-01365-6 PMC9095544 34977960

[jnr25257-bib-0085] Tan, Y. , Fujita, Y. , Qu, Y. , Chang, L. , Pu, Y. , Wang, S. , Wang, X. , & Hashimoto, K. (2020). Phencyclidine‐induced cognitive deficits in mice are ameliorated by subsequent repeated intermittent administration of (R)‐ketamine, but not (S)‐ketamine: Role of BDNF‐TrkB signaling. Pharmacology Biochemistry and Behavior, 188, 172839. 31866390 10.1016/j.pbb.2019.172839

[jnr25257-bib-0086] Temi, S. , Rudyk, C. , Armstrong, J. , Landrigan, J. A. , Dedek, C. , Salmaso, N. , & Hildebrand, M. E. (2021). Differential expression of GluN2 NMDA receptor subunits in the dorsal horn of male and female rats. Channels, 15(1), 179–192. 10.1080/19336950.2020.1871205 PMC7849732 33509021

[jnr25257-bib-0087] Thomas, A. , Burant, A. , Bui, N. , Graham, D. , Yuva‐Paylor, L. A. , & Paylor, R. (2009). Marble burying reflects a repetitive and perseverative behavior more than novelty‐induced anxiety. Psychopharmacology, 204(2), 361–373. 19189082 10.1007/s00213-009-1466-y PMC2899706

[jnr25257-bib-0088] Tolle, T. , Berthele, A. , Zieglgansberger, W. , Seeburg, P. H. , & Wisden, W. (1993). The differential expression of 16 NMDA and non‐NMDA receptor subunits in the rat spinal cord and in periaqueductal gray. Journal of Neuroscience, 13(12), 5009–5028. 8254358 10.1523/JNEUROSCI.13-12-05009.1993 PMC6576409

[jnr25257-bib-0089] Traynelis, S. F. , Wollmuth, L. P. , McBain, C. J. , Menniti, F. S. , Vance, K. M. , Ogden, K. K. , Hansen, K. B. , Yuan, H. , Myers, S. J. , & Dingledine, R. (2010). Glutamate receptor ion channels: Structure, regulation, and function. Pharmacological Reviews, 62(3), 405–496. 10.1124/pr.109.002451 20716669 PMC2964903

[jnr25257-bib-0090] Turgeon, S. M. , Kim, D. , Pritchard, M. , Salgado, S. , & Thaler, A. (2011). The effects of phencyclidine (PCP) on anxiety‐like behavior in the elevated plus maze and the light–dark exploration test are age dependent, sexually dimorphic, and task dependent. Pharmacology Biochemistry and Behavior, 100(1), 191–198. 21889525 10.1016/j.pbb.2011.08.017

[jnr25257-bib-0091] Turner, E. H. (2019). Esketamine for treatment‐resistant depression: Seven concerns about efficacy and FDA approval. Lancet Psychiatry, 6(12), 977–979. 31680014 10.1016/S2215-0366(19)30394-3

[jnr25257-bib-0092] Usun, Y. , Eybrard, S. , Meyer, F. , & Louilot, A. (2013). Ketamine increases striatal dopamine release and hyperlocomotion in adult rats after postnatal functional blockade of the prefrontal cortex. Behavioural Brain Research, 256, 229–237. 23958806 10.1016/j.bbr.2013.08.017

[jnr25257-bib-0093] van den Buuse, M. , Halley, P. , Hill, R. , Labots, M. , & Martin, S. (2012). Altered N‐methyl‐d‐aspartate receptor function in reelin heterozygous mice: Male‐female differences and comparison with dopaminergic activity. Progress in Neuro‐Psychopharmacology & Biological Psychiatry, 37(2), 237–246. 10.1016/j.pnpbp.2012.02.005 22361156

[jnr25257-bib-0094] White, P. , Schüttler, J. , Shafer, A. , Stanski, D. , Horai, Y. , & Trevor, A. (1985). Comparative pharmacology of the ketamine isomers: Studies in volunteers. British Journal of Anaesthesia, 57(2), 197–203. 3970799 10.1093/bja/57.2.197

[jnr25257-bib-0095] Widman, A. J. , & McMahon, L. L. (2018). Disinhibition of CA1 pyramidal cells by low‐dose ketamine and other antagonists with rapid antidepressant efficacy. Proceedings of the National Academy of Sciences of the United States of America, 115(13), E3007–E3016. 29531088 10.1073/pnas.1718883115 PMC5879689

[jnr25257-bib-0096] Williams, N. R. , Heifets, B. D. , Bentzley, B. S. , Blasey, C. , Sudheimer, K. D. , Hawkins, J. , Lyons, D. M. , & Schatzberg, A. F. (2019). Attenuation of antidepressant and antisuicidal effects of ketamine by opioid receptor antagonism. Molecular Psychiatry, 24(12), 1779–1786. 31467392 10.1038/s41380-019-0503-4

[jnr25257-bib-0097] Williams, N. R. , Heifets, B. D. , Blasey, C. , Sudheimer, K. , Pannu, J. , Pankow, H. , Hawkins, J. , Birnbaum, J. , Lyons, D. M. , Rodriguez, C. I. , & Schatzberg, A. F. (2018). Attenuation of antidepressant effects of ketamine by opioid receptor antagonism. American Journal of Psychiatry, 175(12), 1205–1215. 30153752 10.1176/appi.ajp.2018.18020138 PMC6395554

[jnr25257-bib-0098] Wu, Z. , Yang, Z. , Zhang, M. , Bao, X. , Han, F. , & Li, L. (2018). The role of N‐methyl‐D‐aspartate receptors and metabotropic glutamate receptor 5 in the prepulse inhibition paradigms for studying schizophrenia: Pharmacology, neurodevelopment, and genetics. Behavioural Pharmacology, 29(1), 13–27. 29176430 10.1097/FBP.0000000000000352

[jnr25257-bib-0099] Wyllie, D. , Livesey, M. , & Hardingham, G. (2013). Influence of GluN2 subunit identity on NMDA receptor function. Neuropharmacology, 74, 4–17. 23376022 10.1016/j.neuropharm.2013.01.016 PMC3778433

[jnr25257-bib-0100] Xu, K. , Krystal, J. H. , Ning, Y. , Chen, D. C. , He, H. , Wang, D. , Ke, X. , Zhang, X. , Ding, Y. , Liu, Y. , Gueorguieva, R. , Wang, Z. , Limoncelli, D. , Pietrzak, R. H. , Petrakis, I. L. , Zhang, X. , & Fan, N. (2015). Preliminary analysis of positive and negative syndrome scale in ketamine‐associated psychosis in comparison with schizophrenia. Journal of Psychiatric Research, 61, 64–72. 25560772 10.1016/j.jpsychires.2014.12.012 PMC4445679

[jnr25257-bib-0101] Yamamoto, H. , Kamegaya, E. , Hagino, Y. , Takamatsu, Y. , Sawada, W. , Matsuzawa, M. , Ide, S. , Yamamoto, T. , Mishina, M. , & Ikeda, K. (2017). Loss of GluN2D subunit results in social recognition deficit, social stress, 5‐HT2C receptor dysfunction, and anhedonia in mice. Neuropharmacology, 112, 188–197. 27480795 10.1016/j.neuropharm.2016.07.036

[jnr25257-bib-0102] Yamamoto, H. , Kamegaya, E. , Sawada, W. , Hasegawa, R. , Yamamoto, T. , Hagino, Y. , Takamatsu, Y. , Imai, K. , Koga, H. , & Mishina, M. (2013). Involvement of the N‐methyl‐d‐aspartate receptor GluN2D subunit in phencyclidine‐induced motor impairment, gene expression, and increased Fos immunoreactivity. Molecular Brain, 6(1), 1–16. 24330819 10.1186/1756-6606-6-56 PMC3878647

[jnr25257-bib-0103] Yamamoto, T. , Nakayama, T. , Yamaguchi, J. , Matsuzawa, M. , Mishina, M. , Ikeda, K. , & Yamamoto, H. (2016). Role of the NMDA receptor GluN2D subunit in the expression of ketamine‐induced behavioral sensitization and region‐specific activation of neuronal nitric oxide synthase. Neuroscience Letters, 610, 48–53. 10.1016/j.neulet.2015.10.049 26520463

[jnr25257-bib-0104] Yang, C. , Han, M. , Zhang, J.‐C. , Ren, Q. , & Hashimoto, K. (2016). Loss of parvalbumin‐immunoreactivity in mouse brain regions after repeated intermittent administration of esketamine, but not R‐ketamine. Psychiatry Research, 239, 281–283. 27043274 10.1016/j.psychres.2016.03.034

[jnr25257-bib-0105] Yang, C. , Shirayama, Y. , Zhang, J. , Ren, Q. , Yao, W. , Ma, M. , Dong, C. , & Hashimoto, K. (2015). R‐ketamine: A rapid‐onset and sustained antidepressant without psychotomimetic side effects. Translational Psychiatry, 5(9), e632. 26327690 10.1038/tp.2015.136 PMC5068814

[jnr25257-bib-0106] Zhang, B. , Yang, X. , Ye, L. , Liu, R. , Ye, B. , Du, W. , Shen, F. , Li, Q. , Guo, F. , & Liu, J. (2021). Ketamine activated glutamatergic neurotransmission by GABAergic disinhibition in the medial prefrontal cortex. Neuropharmacology, 194, 108382. 33144117 10.1016/j.neuropharm.2020.108382

[jnr25257-bib-0107] Zhang, J.‐C. , Li, S.‐X. , & Hashimoto, K. (2014). R (−)‐ketamine shows greater potency and longer lasting antidepressant effects than S (+)‐ketamine. Pharmacology Biochemistry and Behavior, 116, 137–141. 24316345 10.1016/j.pbb.2013.11.033

[jnr25257-bib-0108] Zhang, Y. , Ye, F. , Zhang, T. , Lv, S. , Zhou, L. , Du, D. , Lin, H. , Guo, F. , Luo, C. , & Zhu, S. (2021). Structural basis of ketamine action on human NMDA receptors. Nature, 596(7871), 301–305. 34321660 10.1038/s41586-021-03769-9

