# Supplementary material for: Understanding the role of the NMDA receptor subunit, GluN2D, in mediating NMDA receptor antagonist‐induced behavioral disruptions in male and female mice
Source: J Neurosci Res. 2023 Oct 10;102(1):e25257. doi: 10.1002/jnr.25257 (PMC10953441; doi:10.1002/jnr.25257)
Supplement: Supplementary file 1 — FIGURE S1 Body weight. GluN2D‐KO mice weighed less than WT controls independent of sex at (a) week 10 and (b) week 16. As expected, male mice were overall heavier than female mice at both time points. All data presented as mean ± SEM; ****p < .0001 main effect of genotype, #### p < .0001 main effect of sex. [file JNR-102-0-s001.pdf]

Supplementary Figure 1

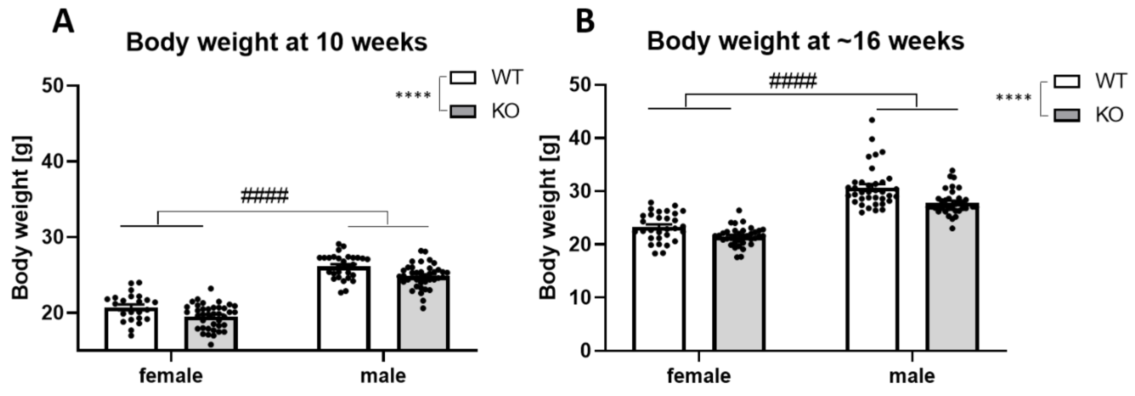

Supplementary Figure 1. Body weight *GluN2D-KO mice weighed less than WT controls independent of sex at (A) week 10 and (B) week ~16. As expected, male mice were overall heavier than female mice at both time points. All data presented as mean ± SEM; ****p < 0.0001-main effect of genotype, ####p < 0.0001-main effect of sex*

Body weight

Body weight was taken at week 10 (just before behavioural experiments) and week ~16 (after the completion of all experiments) from Cohort 1 (Supplemental Fig. 1). For body weight at 10 weeks, a 2-way ANOVA showed a significant main effect of sex ($F(1, 127) = 329.7$; $p < 0.0001$) and genotype ($F(1, 127) = 17.9$; $p < 0.0001$) indicating that KO mice weighed less than their WT controls, independent of sex and, as expected, all male mice were heavier than female mice. No sex x genotype interaction was found. The same trend was found for the 16-week time-point. A 2-way ANOVA revealed a significant main effect of sex ($F(1, 134) = 212.6$; $p < 0.0001$) and genotype ($F(1, 127) = 24.4$; $p < 0.0001$), again, indicating that KO mice were smaller than their WT controls, independent of sex and all male mice were heavier than female mice. Overall, this shows that KO mice weighed less than WT mice throughout the experimental phase from week 10 to week 16.
