# Supplementary material for: Understanding the role of the NMDA receptor subunit, GluN2D, in mediating NMDA receptor antagonist‐induced behavioral disruptions in male and female mice
Source: J Neurosci Res. 2023 Oct 10;102(1):e25257. doi: 10.1002/jnr.25257 (PMC10953441; doi:10.1002/jnr.25257)
Supplement: Supplementary file 2 — TABLE S1 Three‐way ANOVA results for locomotor scoring assay. TABLE S2 Two‐way ANOVA results for locomotor scoring assay. TABLE S3 Results from Tukey's multiple comparisons test for Locomotor Scoring Assay. TABLE S4 Three‐way ANOVA results for locomotor test. TABLE S5 Two‐way ANOVA results for locomotor test. TABLE S6 Results from Sidak's multiple comparisons test for locomotor activity. TABLE S7 Results from Sidak's multiple comparisons test for locomotor activity. TABLE S8 Three‐way ANOVA results from open field test (OFT). TABLE S9 Two‐way ANOVA results from open field test (OFT). TABLE S10 Results from Sidak's multiple comparisons test for open field test (OFT). TABLE S11 Three‐way ANOVA results from elevated‐plus maze (EPM). TABLE S12 Two‐way ANOVA results from elevated‐plus maze (EPM). TABLE S13 Results from Sidak's multiple comparisons test for the elevated‐plus maze (EPM). TABLE S14 Results from Sidak's multiple comparisons test for the elevated‐plus maze (EPM). TABLE S15 Three‐way ANOVA results for the Y‐maze. TABLE S16 Two‐way ANOVA results for the Y‐maze. TABLE S17 Results from Dunnett's multiple comparisons test for the Y‐maze. TABLE S19 Results from Dunnett's multiple comparisons test for the Novel object recognition task (NORT). TABLE S18 Three‐way ANOVA results for the Novel object recognition task (NORT). [file JNR-102-0-s002.docx]

# Supplementary Material

| **Table 1: Three-way ANOVA results for locomotor scoring assay** | | | | | | | | | |
| --- | --- | --- | --- | --- | --- | --- | --- | --- | --- |
|  | Ataxia | | | Stereotypy | | | Catalepsy | | |
|  | P-value | P value summary | F (DFn, DFd) | P-value | P value summary | F (DFn, DFd) | P-value | P value summary | F (DFn, DFd) |
| Treatment | <0.0001 | **** | F (2, 113) = 209.6 | <0.0001 | **** | F (2, 113) = 37.35 | <0.0001 | **** | F (2, 113) = 27.35 |
| Sex | 0.2448 | ns | F (1, 113) = 1.367 | 0.0481 | * | F (1, 113) = 3.992 | 0.2011 | ns | F (1, 113) = 1.653 |
| Genotype | 0.9908 | ns | F (1, 113) = 0.0001322 | 0.2328 | ns | F (1, 113) = 1.439 | 0.3494 | ns | F (1, 113) = 0.8829 |
| Treatment x Sex | 0.7098 | ns | F (2, 113) = 0.3438 | 0.0166 | * | F (2, 113) = 4.252 | 0.2331 | ns | F (2, 113) = 1.475 |
| Treatment x Genotype | 0.9875 | ns | F (2, 113) = 0.01258 | 0.3534 | ns | F (2, 113) = 1.050 | 0.6419 | ns | F (2, 113) = 0.4451 |
| Sex x Genotype | 0.2126 | ns | F (1, 113) = 1.571 | 0.7609 | ns | F (1, 113) = 0.09304 | 0.2899 | ns | F (1, 113) = 1.130 |
| Treatment x Sex x Genotype | 0.566 | ns | F (2, 113) = 0.5720 | 0.888 | ns | F (2, 113) = 0.1189 | 0.2768 | ns | F (2, 113) = 1.299 |

| **Table 2: Two-way ANOVA results for locomotor scoring assay** | | | | | | | | | | | | |
| --- | --- | --- | --- | --- | --- | --- | --- | --- | --- | --- | --- | --- |
|  | Ataxia consolidated sexes | | | Female Stereotypy | | | Male Stereotypy | | | Catalepsy consolidated sexes | | |
|  | P-value | P value summary | F (DFn, DFd) | P-value | P value summary | F (DFn, DFd) | P-value | P value summary | F (DFn, DFd) | P-value | P value summary | F (DFn, DFd) |
| Interaction | 0.952 | ns | F (2, 119) = 0.04920 | 0.1868 | ns | F (2, 48) = 1.738 | 0.8203 | ns | F (2, 65) = 0.1987 | 0.8164 | ns | F (2, 119) = 0.2031 |
| Treatment | <0.0001 | **** | F (2, 119) = 221.0 | <0.0001 | **** | F (2, 48) = 18.30 | <0.0001 | **** | F (2, 65) = 26.79 | <0.0001 | **** | F (2, 119) = 30.16 |
| Genotype | 0.8281 | ns | F (1, 119) = 0.04736 | 0.1522 | ns | F (1, 48) = 2.117 | 0.5648 | ns | F (1, 65) = 0.3348 | 0.4745 | ns | F (1, 119) = 0.5146 |

| **Table 3: Results from Tukey's multiple comparisons test for Locomotor Scoring Assay** | | | | | | | | | | | | |
| --- | --- | --- | --- | --- | --- | --- | --- | --- | --- | --- | --- | --- |
|  | Ataxia consolidated sexes | | | Female Stereotypy | | | Male Stereotypy | | | Catalepsy consolidated sexes | | |
|  | P-value | P value summary | DF | P-value | P value summary | DF | P-value | P value summary | DF | P-value | P value summary | DF |
| R-norket vs. S-ket | <0.0001 | **** | 119 | 0.1621 | ns | 48 | <0.0001 | **** | 65 | <0.0001 | **** | 119 |
| R-norket vs. PCP | <0.0001 | **** | 119 | 0.0005 | *** | 48 | 0.0276 | * | 65 | 0.9666 | ns | 119 |
| S-ket vs. PCP | <0.0001 | **** | 119 | <0.0001 | **** | 48 | <0.0001 | **** | 65 | <0.0001 | **** | 119 |

| **Table 4: Three-way ANOVA results for locomotor test** | | | | | | | | | | | | | | | |
| --- | --- | --- | --- | --- | --- | --- | --- | --- | --- | --- | --- | --- | --- | --- | --- |
|  | Locomotor: drug challenge average distance | | | Locomotor activity: Saline | | | Locomotor activity: R-norket | | | Locomotor activity: S-ket | | | Locomotor activity: PCP | | |
|  | P-value | P value summary | F (DFn, DFd) | P-value | P value summary | F (DFn, DFd) | P-value | P value summary | F (DFn, DFd) | P-value | P value summary | F (DFn, DFd) | P-value | P value summary | F (DFn, DFd) |
| Treatment/Time | 0.0001 | *** | F (3, 103) = 7.713 | <0.0001 | **** | F (4.012, 132.4) = 59.62 | <0.0001 | **** | F (2.702, 94.58) = 49.86 | <0.0001 | **** | F (4.116, 139.9) = 46.95 | <0.0001 | **** | F (3.304, 118.9) = 25.59 |
| Sex | 0.088 | ns | F (1, 103) = 2.967 | 0.6947 | ns | F (1, 33) = 0.1568 | 0.7223 | ns | F (1, 35) = 0.1283 | 0.0703 | ns | F (1, 34) = 3.492 | 0.3551 | ns | F (1, 36) = 0.8776 |
| Genotype | <0.0001 | **** | F (1, 103) = 32.62 | 0.014 | * | F (1, 33) = 6.739 | 0.2462 | ns | F (1, 35) = 1.391 | 0.0106 | * | F (1, 34) = 7.315 | 0.0019 | ** | F (1, 36) = 11.19 |
| Treatment/Time x Sex | 0.1775 | ns | F (3, 103) = 1.673 | 0.0805 | ns | F (8, 264) = 1.783 | 0.9987 | ns | F (8, 280) = 0.1140 | 0.292 | ns | F (8, 272) = 1.212 | 0.0003 | *** | F (8, 288) = 3.794 |
| Treatment/Time x Genotype | 0.0429 | * | F (3, 103) = 2.814 | 0.0002 | *** | F (8, 264) = 4.011 | 0.0033 | ** | F (8, 280) = 2.968 | <0.0001 | **** | F (8, 272) = 4.433 | <0.0001 | **** | F (8, 288) = 4.539 |
| Sex x Genotype | 0.0247 | * | F (1, 103) = 5.195 | 0.3188 | ns | F (1, 33) = 1.025 | 0.5402 | ns | F (1, 35) = 0.3827 | 0.2092 | ns | F (1, 34) = 1.638 | 0.6039 | ns | F (1, 36) = 0.2740 |
| Treatment/Time x Sex x Genotype | 0.9044 | ns | F (3, 103) = 0.1879 | 0.3564 | ns | F (8, 264) = 1.110 | 0.4898 | ns | F (8, 280) = 0.9326 | 0.563 | ns | F (8, 272) = 0.8458 | 0.0072 | ** | F (8, 288) = 2.690 |

| **Table 5: Two-way ANOVA results for locomotor test** | | | | | | | | | | | | | | | |
| --- | --- | --- | --- | --- | --- | --- | --- | --- | --- | --- | --- | --- | --- | --- | --- |
|  | Locomotor: drug challenge average distance consolidated sexes | | | Locomotor activity: Saline consolidated sexes | | | Locomotor activity: R-norket consolidated sexes | | | Locomotor activity: S-ket | | | Locomotor activity: PCP females | | |
|  | P-value | P value summary | F (DFn, DFd) | P-value | P value summary | F (DFn, DFd) | P-value | P value summary | F (DFn, DFd) | P-value | P value summary | F (DFn, DFd) | P-value | P value summary | F (DFn, DFd) |
| Interaction | 0.0619 | ns | F (3, 111) = 2.517 | 0.0003 | *** | F (8, 280) = 3.790 | 0.003 | ** | F (8, 296) = 3.003 | <0.0001 | **** | F (8, 288) = 4.423 | 0.0024 | ** | F (8, 136) = 3.195 |
| Treatment/Time | 0.0004 | *** | F (3, 111) = 6.669 | <0.0001 | **** | F (3.954, 138.4) = 58.86 | <0.0001 | **** | F (2.777, 102.8) = 51.37 | <0.0001 | **** | F (4.308, 155.1) = 47.52 | <0.0001 | **** | F (3.195, 54.32) = 23.57 |
| Genotype | <0.0001 | **** | F (1, 111) = 31.11 | 0.0116 | * | F (1, 35) = 7.104 | 0.2387 | ns | F (1, 37) = 1.434 | 0.0115 | * | F (1, 36) = 7.089 | 0.0705 | ns | F (1, 17) = 3.723 |

| **Table 6: Results from Sidak's multiple comparisons test for locomotor activity** | | | |  |
| --- | --- | --- | --- | --- |
|  | Locomotor: drug challenge average distance consolidated sexes | | | |
| WT - KO | P-value | P value summary | DF | |
| veh | 0.4915 | ns | 111 | |
| R-Ket | 0.0496 | * | 111 | |
| S-Ket | 0.0951 | ns | 111 | |
| PCP | <0.0001 | **** | 111 | |

| **Table 7: Results from Sidak's multiple comparisons test for locomotor activity** | | | | | | | | | | | |  | **Fisher's LSD test** | | |  | |
| --- | --- | --- | --- | --- | --- | --- | --- | --- | --- | --- | --- | --- | --- | --- | --- | --- | --- |
|  | Locomotor activity: Saline consolidated sexes | | | Locomotor activity: R-norket consolidated sexes | | | Locomotor activity: S-ket consolidated sexes | | | Locomotor activity: PCP females | | | Locomotor activity: PCP males | | | |  |
| WT - KO | P-value | P value summary | DF | P-value | P value summary | DF | P-value | P value summary | DF | P-value | P value summary | DF | P-value | P value summary | DF | |  |
| 20 | 0.0088 | ** | 34.94 | 0.0109 | * | 35.9 | 0.0063 | ** | 35.78 | 0.3864 | ns | 9.356 | 0.0201 | * | 38 | |  |
| 40 | 0.03 | * | 34.95 | 0.126 | ns | 35.36 | 0.0566 | ns | 35.96 | 0.3718 | ns | 9.203 | 0.0232 | * | 38 | |  |
| 60 | 0.0103 | * | 34.82 | 0.1587 | ns | 35.88 | 0.0774 | ns | 34.35 | 0.3445 | ns | 12.37 | 0.0811 | ns | 17.26 | |  |
| 80 | 0.0384 | * | 33.85 | 0.8635 | ns | 30.5 | 0.0696 | ns | 32.58 | 0.2167 | ns | 15.31 | 0.0088 | ** | 15.57 | |  |
| 100 | 0.8799 | ns | 32.84 | 0.9996 | ns | 29.06 | 0.2676 | ns | 31.23 | 0.8073 | ns | 10.27 | 0.0175 | * | 14.28 | |  |
| 120 | 0.769 | ns | 34.56 | >0.9999 | ns | 35.73 | 0.9194 | ns | 34.11 | 0.7245 | ns | 9.428 | 0.0158 | * | 12.62 | |  |
| 140 | 0.9682 | ns | 34.23 | >0.9999 | ns | 30.39 | >0.9999 | ns | 35.54 | >0.9999 | ns | 15.71 | 0.0661 | ns | 13.73 | |  |
| 160 | 0.8483 | ns | 34.97 | >0.9999 | ns | 36.81 | 0.9131 | ns | 35.76 | 0.9469 | ns | 12.92 | 0.2694 | ns | 11.67 | |  |
| 180 | >0.9999 | ns | 34.62 | >0.9999 | ns | 36.99 | >0.9999 | ns | 35.93 | 0.9944 | ns | 13.32 | 0.1891 | ns | 12.15 | |  |

| **Table 8: Three-way ANOVA results from open field test (OFT)** | | | |
| --- | --- | --- | --- |
|  | OFT: 2 hours post drug challenge | | |
|  | P-value | P value summary | F (DFn, DFd) |
| drug | 0.0077 | ** | F (3, 139) = 4.133 |
| sex | 0.4092 | ns | F (1, 139) = 0.6853 |
| genotype | 0.6723 | ns | F (1, 139) = 0.1797 |
| drug x sex | 0.5822 | ns | F (3, 139) = 0.6532 |
| drug x genotype | 0.163 | ns | F (3, 139) = 1.733 |
| sex x genotype | 0.4197 | ns | F (1, 139) = 0.6551 |
| drug x sex x genotype | 0.2348 | ns | F (3, 139) = 1.437 |

| **Table 9: Two-way ANOVA results from open field test (OFT)** | | | | | | | | | |
| --- | --- | --- | --- | --- | --- | --- | --- | --- | --- |
|  | OFT: first 60 mins | | | Female OFT: 2 hours post drug challenge | | | Male OFT: 2 hours post drug challenge | | |
|  | P-value | P value summary | F (DFn, DFd) | P-value | P value summary | F (DFn, DFd) | P-value | P value summary | F (DFn, DFd) |
| Interaction | 0.1883 | ns | F (1, 149) = 1.747 | 0.6475 | ns | F (3, 69) = 0.5536 | 0.0399 | * | F (3, 70) = 2.922 |
| sex/drug | 0.0026 | ** | F (1, 149) = 9.383 | 0.5168 | ns | F (3, 69) = 0.7664 | 0.0056 | ** | F (3, 70) = 4.570 |
| genotype | 0.0005 | *** | F (1, 149) = 12.73 | 0.7987 | ns | F (1, 69) = 0.06554 | 0.353 | ns | F (1, 70) = 0.8741 |

| **Table 10: Results from Sidak's multiple comparisons test for open field test (OFT)** | | | |
| --- | --- | --- | --- |
|  | Male OFT: 2 hours post drug challenge | | |
|  | P-value | P value summary | DF |
| saline:M WT vs. saline:M KO | 0.2455 | ns | 70 |
| saline:M WT vs. R-norket:M WT | 0.0616 | ns | 70 |
| saline:M WT vs. R-norket:M KO | 0.8055 | ns | 70 |
| saline:M WT vs. S-ket:M WT | 0.0023 | ** | 70 |
| saline:M WT vs. S-ket:M KO | 0.0923 | ns | 70 |
| saline:M WT vs. PCP:M WT | 0.0015 | ** | 70 |
| saline:M WT vs. PCP:M KO | 0.0831 | ns | 70 |

| **Table 11: Three-way ANOVA results from elevated-plus maze (EPM)** | | | | | | |
| --- | --- | --- | --- | --- | --- | --- |
|  | EPM: Open/closed arms | | | EPM: Latency to closed arm | | |
|  | P-value | P value summary | F (DFn, DFd) | P-value | P value summary | F (DFn, DFd) |
| drug | <0.0001 | **** | F (3, 128) = 9.290 | 0.1947 | ns | F (3, 128) = 1.591 |
| sex | 0.0002 | *** | F (1, 128) = 14.86 | 0.2168 | ns | F (1, 128) = 1.541 |
| genotype | 0.3812 | ns | F (1, 128) = 0.7722 | 0.051 | ns | F (1, 128) = 3.881 |
| drug x sex | 0.0186 | * | F (3, 128) = 3.452 | 0.0915 | ns | F (3, 128) = 2.197 |
| drug x genotype | 0.2951 | ns | F (3, 128) = 1.248 | 0.0366 | * | F (3, 128) = 2.921 |
| sex x genotype | 0.0235 | * | F (1, 128) = 5.258 | 0.353 | ns | F (1, 128) = 0.8689 |
| drug x sex x genotype | 0.2604 | ns | F (3, 128) = 1.352 | 0.0002 | *** | F (3, 128) = 6.922 |

| **Table 12: Two-way ANOVA results from elevated-plus maze (EPM)** | | | | | | | | | | | | |
| --- | --- | --- | --- | --- | --- | --- | --- | --- | --- | --- | --- | --- |
|  | Females EPM: Open/closed arms | | | Males EPM: Open/closed arms | | | Females EPM: Latency to closed arms | | | Males EPM: Latency to closed arms | | |
|  | P-value | P value summary | F (DFn, DFd) | P-value | P value summary | F (DFn, DFd) | P-value | P value summary | F (DFn, DFd) | P-value | P value summary | F (DFn, DFd) |
| Interaction | 0.0231 | * | F (3, 66) = 3.387 | 0.4488 | ns | F (3, 62) = 0.8953 | <0.0001 | **** | F (3, 67) = 8.744 | 0.03 | * | F (3, 61) = 3.186 |
| sex/drug | 0.0154 | * | F (3, 66) = 3.730 | 0.0011 | ** | F (3, 62) = 6.056 | 0.6808 | ns | F (3, 67) = 0.5040 | 0.0714 | ns | F (3, 61) = 2.457 |
| genotype | 0.0512 | ns | F (1, 66) = 3.945 | 0.1033 | ns | F (1, 62) = 2.733 | 0.0114 | * | F (1, 67) = 6.775 | 0.5436 | ns | F (1, 61) = 0.3730 |

| **Table 13: Results from Sidak's multiple comparisons test for the elevated-plus maze (EPM)** | | | | | | |
| --- | --- | --- | --- | --- | --- | --- |
|  | Females EPM: Open/closed arms | | | Males EPM: Open/closed arms | | |
|  | P-value | P value summary | DF | P-value | P value summary | DF |
| saline vs. R-norket | 0.9832 | ns | 66 | 0.9805 | ns | 62 |
| saline vs. S-ket | 0.9866 | ns | 66 | 0.993 | ns | 62 |
| saline vs. PCP | 0.1217 | ns | 66 | 0.0018 | ** | 62 |
| R-norket vs. S-ket | 0.6251 | ns | 66 | 0.9996 | ns | 62 |
| R-norket vs. PCP | 0.354 | ns | 66 | 0.0054 | ** | 62 |
| S-ket vs. PCP | 0.0124 | * | 66 | 0.0053 | ** | 62 |

| **Table 14: Results from Sidak's multiple comparisons test for the elevated-plus maze (EPM)** | | | | | | |
| --- | --- | --- | --- | --- | --- | --- |
|  | Females EPM: Latency to closed arms | | | Males EPM: Latency to closed arms | | |
|  | P-value | P value summary | F (DFn, DFd) | P-value | P value summary | F (DFn, DFd) |
| WT |  |  |  |  |  |  |
| saline vs. R-norket | 0.2212 | ns | 67 | 0.224 | ns | 61 |
| saline vs. S-ket | >0.9999 | ns | 67 | 0.0058 | ** | 61 |
| saline vs. PCP | 0.9841 | ns | 67 | 0.4778 | ns | 61 |
| R-norket vs. S-ket | 0.2866 | ns | 67 | 0.3163 | ns | 61 |
| R-norket vs. PCP | 0.0491 | * | 67 | 0.9719 | ns | 61 |
| S-ket vs. PCP | 0.9265 | ns | 67 | 0.1788 | ns | 61 |
| KO |  |  |  |  |  |  |
| saline vs. R-norket | 0.0097 | ** | 67 | 0.9144 | ns | 61 |
| saline vs. S-ket | >0.9999 | ns | 67 | 0.9997 | ns | 61 |
| saline vs. PCP | >0.9999 | ns | 67 | 0.6188 | ns | 61 |
| R-norket vs. S-ket | 0.0062 | ** | 67 | 0.9487 | ns | 61 |
| R-norket vs. PCP | 0.0026 | ** | 67 | 0.3139 | ns | 61 |
| S-ket vs. PCP | >0.9999 | ns | 67 | 0.6001 | ns | 61 |

| **Table 15: Three-way ANOVA results for the Y-maze** | | | |
| --- | --- | --- | --- |
|  | Y-maze: Discrimination Ratio | | |
|  | P-value | P value summary | F (DFn, DFd) |
| drug | 0.4607 | ns | F (3, 139) = 0.8654 |
| sex | 0.7554 | ns | F (1, 139) = 0.09743 |
| genotype | 0.4357 | ns | F (1, 139) = 0.6110 |
| drug x sex | 0.0396 | * | F (3, 139) = 2.852 |
| drug x genotype | 0.5776 | ns | F (3, 139) = 0.6606 |
| sex x genotype | 0.7224 | ns | F (1, 139) = 0.1267 |
| drug x sex x genotype | 0.5982 | ns | F (3, 139) = 0.6278 |

| **Table 16: Two-way ANOVA results for the Y-maze** | | | | | | |
| --- | --- | --- | --- | --- | --- | --- |
|  | Female Y-maze: Discrimination Ratio | | | Male Y-maze: Discrimination Ratio | | |
|  | P-value | P value summary | F (DFn, DFd) | P-value | P value summary | F (DFn, DFd) |
| Interaction | 0.7365 | ns | F (3, 68) = 0.4238 | 0.4881 | ns | F (3, 71) = 0.8181 |
| drug | 0.0305 | * | F (3, 68) = 3.148 | 0.3768 | ns | F (3, 71) = 1.048 |
| genotype | 0.3687 | ns | F (1, 68) = 0.8190 | 0.7836 | ns | F (1, 71) = 0.07597 |

| **Table 17: Results from Dunnett's multiple comparisons test for the Y-maze** | | | |  |
| --- | --- | --- | --- | --- |
|  | Female Y-maze: Discrimination Ratio | | |  |
|  | P-value | P value summary | DF |  |
| saline vs. R-norket | 0.9964 | ns | 68 |  |
| saline vs. S-ket | 0.0387 | * | 68 |  |
| saline vs. PCP | 0.9547 | ns | 68 |  |

| **Table 18: Three-way ANOVA results for the Novel object recognition task (NORT)** | | | |
| --- | --- | --- | --- |
|  | NORT: Preference Ratio | | |
|  | P-value | P value summary | F (DFn, DFd) |
| drug | 0.0057 | ** | F (3, 131) = 4.381 |
| sex | 0.4788 | ns | F (1, 131) = 0.5044 |
| genotype | 0.601 | ns | F (1, 131) = 0.2748 |
| drug x sex | 0.2818 | ns | F (3, 131) = 1.287 |
| drug x genotype | 0.3791 | ns | F (3, 131) = 1.036 |
| sex x genotype | 0.3146 | ns | F (1, 131) = 1.019 |
| drug x sex x genotype | 0.5593 | ns | F (3, 131) = 0.6906 |

| **Table 19: Results from Dunnett's multiple comparisons test for the Novel object recognition task (NORT)** | | | |
| --- | --- | --- | --- |
|  | NORT: Preference Ratio | | |
|  | P-value | P value summary | DF |
| saline vs. R-norket | 0.764 | ns | 131 |
| saline vs. S-Ket | 0.024 | * | 131 |
| saline vs. PCP | 0.884 | ns | 131 |
